# The joint association of the combined triglyceride-glucose index and atherogenic index of plasma with hypertension on stroke risk across different glycemic status: a prospective cohort study

**DOI:** 10.1186/s12933-026-03080-9

**Published:** 2026-03-26

**Authors:** Xuelun Zou, Chang Zhou, Ruining Zhou, Ting Zhu, Jieyu Zhao, Wenxiang Qing, Jiawei Xie, Rili Yu, Fan Zhang, Jin Li

**Affiliations:** 1 Department of Anesthesiology, Third Xiangya Hospital, Central South University, Changsha, Hunan 410013 People’s Republic of China; 2 Department of Neurology, Xiangya Hospital, Central South University, Changsha, Hunan 410008 People’s Republic of China; 3 Department of Oncology, Third Xiangya Hospital, Central South University, Changsha, Hunan 410008 People’s Republic of China

**Keywords:** Stroke, TyG-AIP, Hypertension, Insulin resistance, Prospective cohort

## Abstract

**Background:**

The triglyceride-glucose index (TyG) and the atherogenic index of plasma (AIP) are well-established indicators of insulin resistance and lipid metabolism, respectively, and both are associated with stroke risk. However, the joint impact of TyG and AIP—expressed as their product (TyG-AIP)—and its longitudinal trajectory on stroke risk have not been investigated. Moreover, it remains unclear whether TyG-AIP interacts synergistically with hypertension to improve stroke risk prediction.

**Methods:**

This prospective cohort study included 5786 participants, categorized into dysglycemia (PDM, n = 3,490) and normoglycemia (NDM, n = 2,296) groups. TyG-AIP was calculated as the product of TyG and AIP. K-means clustering was applied to identify distinct patterns of TyG-AIP change between the two measurement points. Multivariable Cox proportional hazards models, restricted cubic splines, and receiver operating characteristic (ROC) analyses evaluated associations and predictive performance.

**Results:**

Over 8 years of follow-up, 460 incident stroke cases occurred. Higher TyG-AIP levels were independently associated with an increased risk of stroke (per SD increase: HR = 1.35, 95% CI 1.21–1.51; *P* < 0.001), with a stronger effect among those with dysglycemia (HR = 1.54, 95% CI 1.21–1.95; *P* < 0.001). A nonlinear association was observed (*P* for nonlinearity = 0.002). TyG-AIP synergistically interacted with hypertension, and individuals with both high TyG-AIP and hypertension had the greatest risk (HR = 2.89, 95% CI 2.22–3.76). The “high-and-declining” TyG-AIP trajectory conferred the highest stroke risk in the PDM group (HR = 2.26, 95% CI 1.62–3.15; *P* < 0.001). ROC analysis showed that a model combining TyG-AIP with hypertension (AUC = 0.643) provided improved discrimination compared to hypertension alone (AUC = 0.571).

**Conclusions:**

TyG-AIP is associated with increased stroke risk, particularly in dysglycemic individuals, and exhibits joint effects with hypertension. The integration of TyG-AIP assessment with hypertension status enhances risk stratification, supporting comprehensive management of both metabolic and hemodynamic factors in stroke prevention.

**Graphical abstract:**

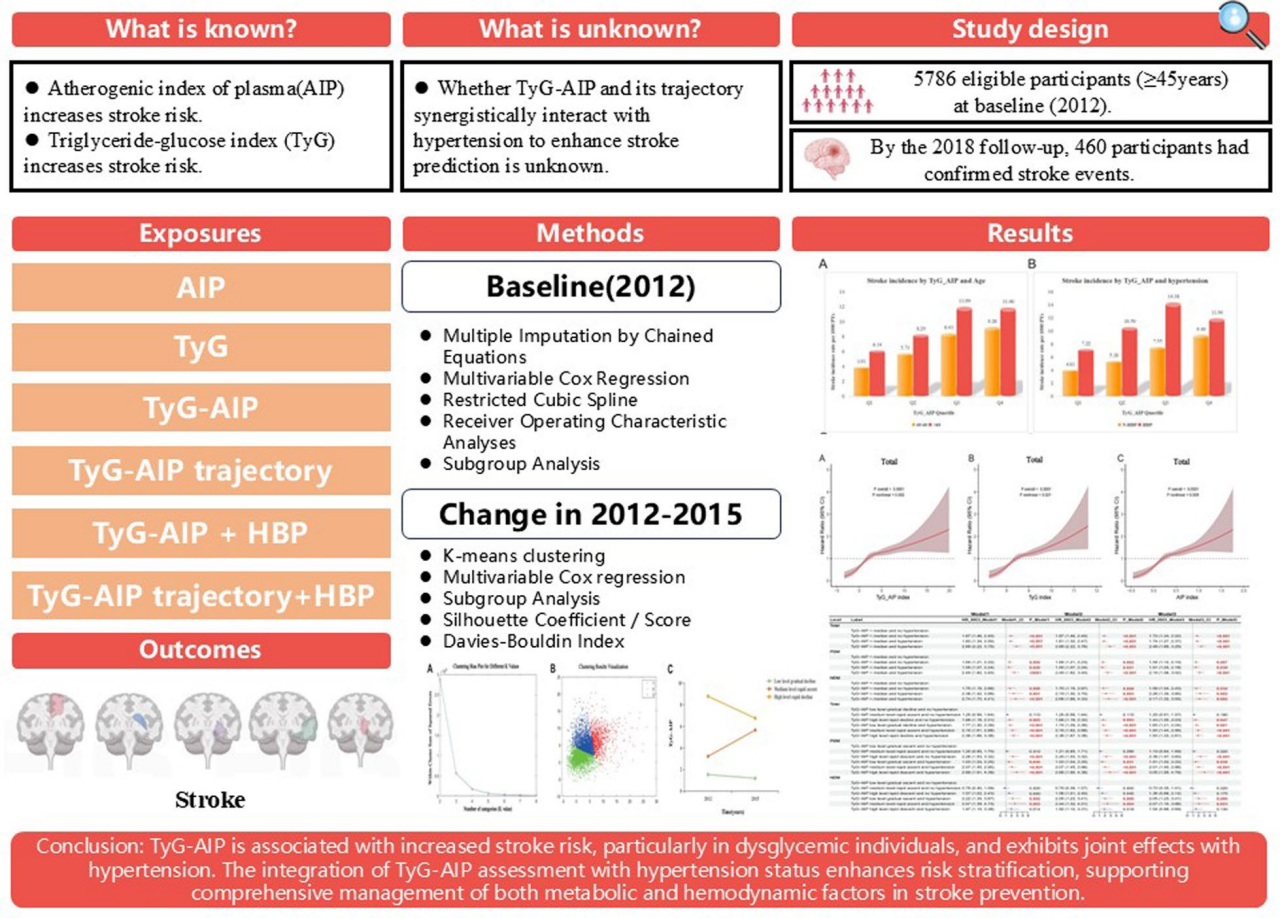

**Supplementary Information:**

The online version contains supplementary material available at 10.1186/s12933-026-03080-9.

## Introduction

Stroke represents a major global public health challenge, with China bearing a particularly heavy burden. Globally, the annual incidence of stroke is estimated at 11.9 million (10.7–13.2 million), and the prevalence reaches 93.8 million (89.0–99.3 million) cases [1]. According to the Global Burden of Disease 2021 data, stroke ranks as the third leading cause of death—following ischemic heart disease and COVID-19—accounting for 10.7% (9.8–11.3%) of all deaths, and the fourth leading cause of disability-adjusted life years (DALYs), contributing 5.6% (5.0–6.1%) of total DALYs [1].In China, approximately 17.8 million adults aged ≥ 40 years had experienced a stroke by 2020, resulting in 2.3 million deaths [2]. With population aging and a rising prevalence of metabolic disorders, stroke incidence is expected to increase further, imposing a substantial economic and societal burden [3]. Therefore, identifying modifiable risk factors and enabling early prevention have become critical priorities in public health.

Among numerous risk factors, insulin resistance (IR) is considered a central pathophysiological mechanism in stroke development [4]. IR promotes atherosclerosis by enhancing inflammation, oxidative stress, and endothelial dysfunction [4]. However, the gold standard for IR assessment—the hyperinsulinemic-euglycemic clamp—is impractical for large-scale or clinical application due to its invasiveness and cost [5]. Consequently, reliable and accessible surrogate indicators are needed. The triglyceride-glucose (TyG) index, derived from routine fasting triglyceride and glucose levels, has been validated as a simple and effective marker of IR and a predictor of stroke risk [6, 7,63]. Similarly, the atherogenic index of plasma (AIP), reflecting lipid metabolic imbalance, has shown promise in assessing atherosclerosis severity and plaque progression beyond conventional lipid markers [8].

Despite their established roles, most studies have focused on the independent associations of TyG and AIP with cardiovascular or cerebrovascular risk, whereas their combined effect remains largely unexplored. Pathophysiologically, metabolic stress (reflected by TyG) and lipid abnormalities (reflected by AIP) may synergistically aggravate vascular injury through shared pathways such as chronic inflammation and endothelial dysfunction. Thus, developing a composite indicator integrating these “dual burdens” may improve precision in stroke risk stratification [9, 10]. Based on this rationale, we propose a novel combined index—TyG-AIP—to comprehensively capture an individual’s metabolic and lipid abnormality burden. Thus, TyG-AIP was conceptualized not as a discovery of a novel biomarker, but as an integrated metric to concurrently capture both insulin resistance and atherogenic lipid burden, potentially offering a more robust and practical tool for risk assessment.

Moreover, hypertension remains the most prevalent and modifiable independent risk factor for stroke [11–13], yet evidence is scarce regarding its potential interaction with TyG-AIP in amplifying stroke risk. Therefore, this prospective cohort study aimed to investigate the association between the TyG-AIP index, its change patterns, and stroke incidence, with a particular focus on its joint effect with hypertension. The findings are expected to offer a more refined risk assessment tool for early identification of high-risk individuals and provide evidence to guide targeted, stratified prevention strategies.

## Methods

### Data source and study sample

This study was based on a nationally representative prospective cohort of Chinese adults aged ≥ 45 years [14].The baseline survey was conducted in 2011, followed by biennial follow-up assessments through 2020, resulting in four survey waves [14]. Participants were recruited from 150 counties or districts across 28 provinces using a multistage, stratified, probability-proportional-to-size sampling design that included both urban and rural areas [14]. In each wave, trained interviewers collected data through face-to-face interviews using standardized questionnaires covering demographics, medical history, lifestyle, and social factors [14]. The 2011–2012 wave was used as the baseline, and follow-up was censored at 2018 to minimize the potential impact of the COVID-19 pandemic on endpoint ascertainment. The primary endpoint was incident stroke.

**Fig. 1:**
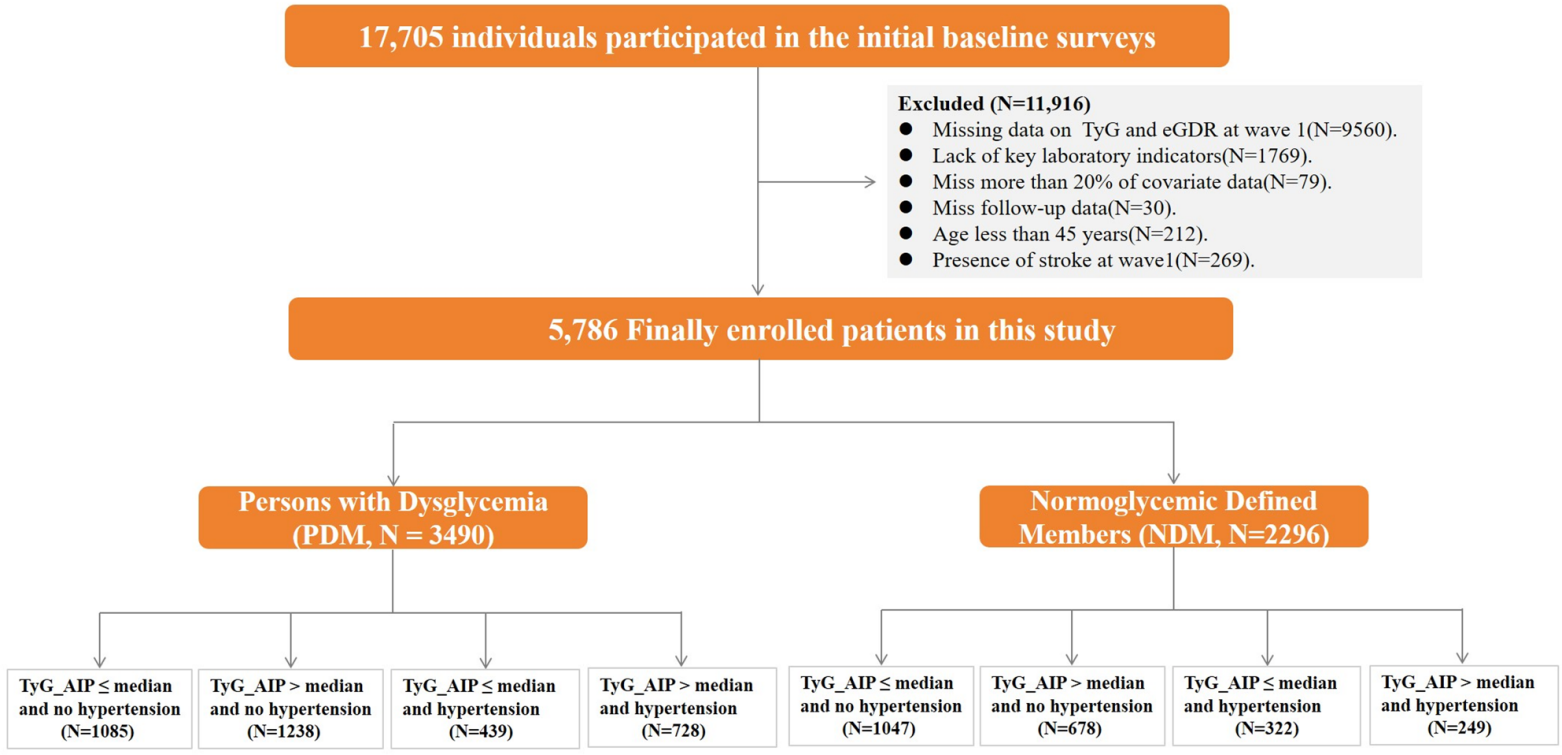
Flowchart of inclusion and exclusion criteria for this study

The baseline cohort included 17,705 participants. After applying inclusion and exclusion criteria consistent with prior studies, 5,789 individuals were retained for analysis (Fig. 1). Participants were classified into a dysglycemia (PDM; n = 3,490) or normoglycemia (NDM; n = 2,296) group based on glycemic status [15]. Dysglycemia was defined as fasting plasma glucose ≥ 6.1 mmol/L, 2 h post-OGTT glucose ≥ 7.8 mmol/L, or HbA1c ≥ 5.7%[15]. Normoglycemia was defined as fasting plasma glucose < 6.1 mmol/L, 2 h postprandial glucose < 7.8 mmol/L, and HbA1c < 5.7% [15].

In this study, we followed the SAGER guidelines for the reporting of sex and gender information. The term ‘sex’ (biological attribute) and ‘male/female’ are used consistently throughout to describe participants (https://ease.org.uk/communities/.

gender-policy-committee/the-sager-guidelines/)[16]. The study adhered to the Declaration of Helsinki and received ethical approval from the Peking University Institutional Review Board (IRB00001052-11015). Written informed consent was obtained from all participants.

### Definition of exposure variables

Fasting venous blood samples were collected by healthcare professionals from the Chinese Center for Disease Control and Prevention following standardized procedures. Laboratory analyses were performed at the Clinical Laboratory Center of You’an Hospital, Capital Medical University. Measurements included plasma glucose, triglycerides (TG), and high-density lipoprotein cholesterol (HDL-C).

Hypertension was defined based on self-reported physician diagnosis and/or meeting at least one of the following criteria: systolic blood pressure ≥ 140 mmHg, diastolic blood pressure ≥ 90 mmHg, or current use of antihypertensive medication [17]. Participants who later denied hypertension or antihypertensive medication use during follow-up were excluded.

The indices were calculated as follows:

- TyG index: ln[(TG (mg/dL) × FBG (mg/dL))/2][18];
- AIP index: log[TG (mmol/L)/HDL-C (mmol/L)][19];
- TyG-AIP composite index: TyG-AIP = TyG × AIP;

Given the established synergistic pathways through which metabolic dysregulation and hypertension jointly promote vascular injury [20–23], we created a combined categorical variable. The primary aims were: (1) to empirically test for a multiplicative interaction between TyG-AIP and hypertension on stroke risk; and (2) to perform an intuitive joint risk stratification that identifies the clinical phenotype of individuals exposed to both a high metabolic burden and hypertension, a subgroup of particular interest for targeted prevention.

Combined TyG-AIP and Hypertension Categories:

TyG-AIP was dichotomized at its median value to define “High” and “Low” groups. Hypertension status was defined as above. A four-level combined variable was then created:

- Q1(Reference) = TyG-AIP ≤ median and no hypertension;
- Q2 = TyG-AIP > median and no hypertension;
- Q3 = TyG-AIP ≤ median and hypertension;
- Q4 = TyG-AIP > median and hypertension [24].

To test for a multiplicative interaction, a product term (continuous TyG-AIP × hypertension status) was included in a Cox regression model, and its significance was assessed using the likelihood ratio test.

### Defining the endpoint event

The primary outcome was the incidence of stroke during the follow-up period, defined as the first occurrence of physician-diagnosed stroke in participants free of stroke at baseline. Stroke cases were identified based on the question, “Have you ever been diagnosed with a stroke by a doctor?” and verified using medical records, treatment information, follow-up confirmations, and medication data [25]. Cases that could not be verified were re-evaluated and excluded.

The observation period extended from baseline to the date of the first confirmed stroke event. For participants without stroke, follow-up time was defined as the interval from baseline to the last completed survey or end of follow-up in 2018.

### Data collection and missing data handling

Baseline data were collected by trained interviewers using structured questionnaires that captured detailed sociodemographic and health-related information. Sociodemographic variables included sex (male or female), age (45–60 years and > 60 years), education level (below high school or high school and above), marital status (married/partnered or unmarried/divorced/widowed), and household registration (rural or urban). Health-related variables included smoking status (current smoker or non-smoker), drinking status (current drinker or non-drinker), and body mass index (BMI). BMI was calculated as weight in kilograms divided by the square of height in meters (kg/m^2^). For the purpose of this study, BMI was categorized as “normal” or “abnormal.” “Normal” BMI was defined as 18.5 kg/m^2^ ≤ BMI < 25.0 kg/m^2^, consistent with the normal weight range of the World Health Organization classification. “Abnormal” BMI was defined as BMI < 18.5 kg/m^2^ (underweight) or BMI ≥ 25.0 kg/m^2^ (overweight and obesity) [26]. Participants self-reported physician-diagnosed chronic conditions, including diabetes, cancer, heart disease, and dyslipidemia. Medication use was identified through affirmative responses to the question: “Do you regularly take anti-diabetic, anti-cancer, heart disease treatment, or lipid-lowering medications as prescribed by a doctor?” In addition, anthropometric measurements (height, weight, waist circumference), physical examinations (blood pressure and pulse), and laboratory indicators—including white blood cell count, hemoglobin, uric acid, blood urea nitrogen, creatinine, triglycerides, C-reactive protein, high-density lipoprotein cholesterol (HDL-C), glycated hemoglobin (HbA1c), fasting blood glucose (FBG), low-density lipoprotein cholesterol (LDL-C), and total cholesterol—were recorded.

Dyslipidemia was defined as meeting any of the following criteria: triglycerides ≥ 2.3 mmol/L, total cholesterol ≥ 6.2 mmol/L, LDL-C ≥ 4.1 mmol/L, HDL-C < 1.0 mmol/L, current use of lipid-lowering therapy, or self-reported physician-diagnosed dyslipidemia[27]. Systolic and diastolic blood pressure were measured twice on the right arm after at least 10 min of rest, and the average value was used for analysis.

Missing data were examined prior to analysis. Variables with missing rates < 5% were imputed using mean or mode substitution, while those exceeding 5% were addressed via multiple imputation under the assumption of missing at random.

### Cluster analysis of TyG-AIP change patterns

**Fig. 2:**
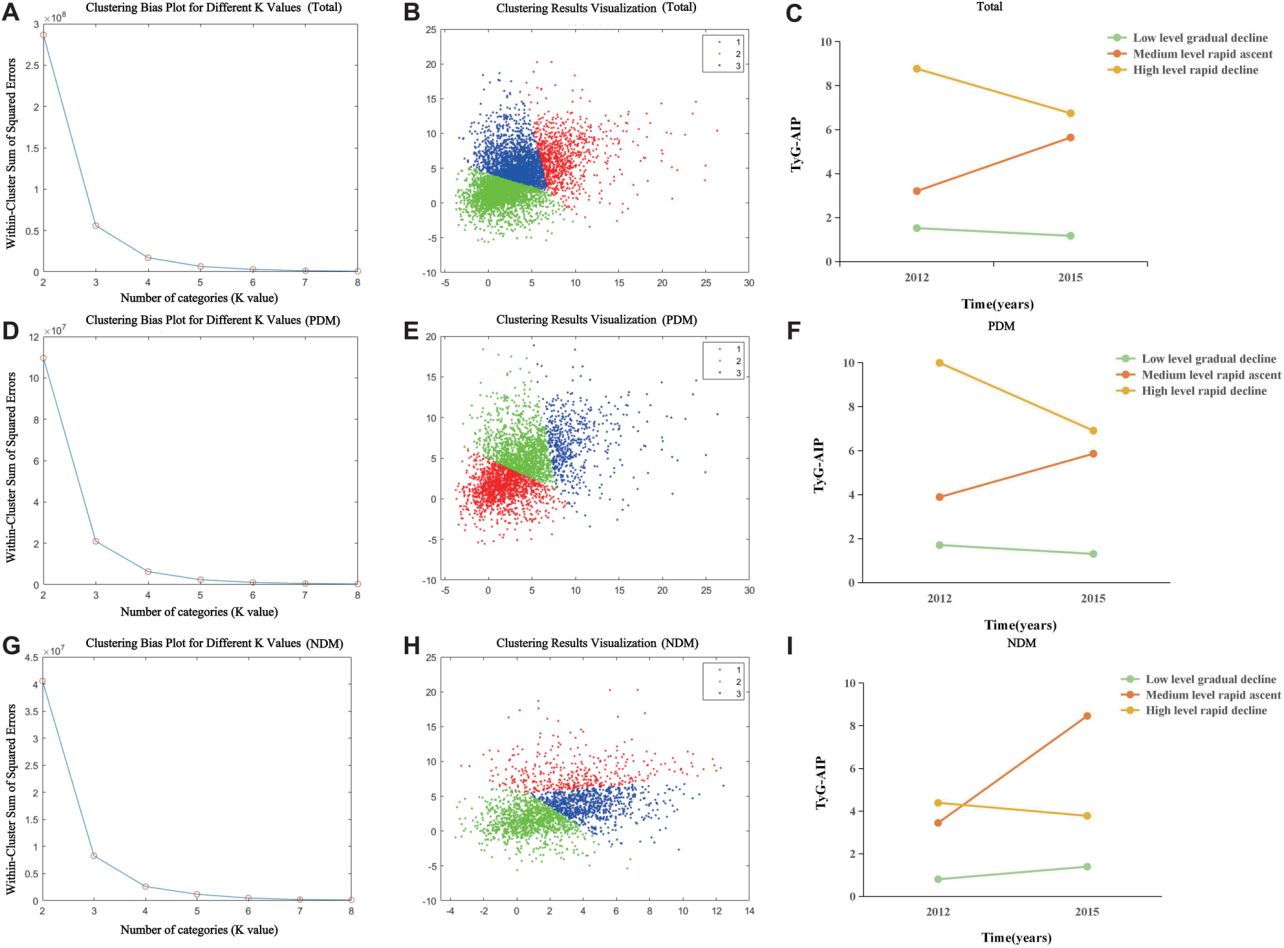
Identification and Characterization of TyG-AIP Trajectory Clusters. This figure illustrates the identification and characterization of TyG-AIP trajectory clusters across three glycemia strata: total population (**A**–**C**), prediabetes (PDM, D-F), and normoglycemia (NDM, G-I). **A**, **D**, **G** The elbow method plots used to determine the optimal number of clusters (K) for K-means clustering within each stratum. **B**, **E**, **H** Scatter plots presenting the K-means clustering results, with TyG-AIP in 2012 plotted against 2015, and points colored by the assigned trajectory cluster. **C**, **F**, **I** Line plots depicting the mean TyG-AIP changes from 2012 to 2015 for each identified trajectory cluster within the respective strata. Three distinct trajectory patterns were identified across all strata: “Low level gradual decline,” “Medium level rapid ascent,” and “High level rapid decline.” The average silhouette coefficient for the overall clustering solution was 0.479. T2D, type 2 diabetes; PDM, prediabetes; NDM, newly diagnosed diabetes

To identify distinct patterns of TyG-AIP change, the TyG-AIP index from 2012 and 2015 was analyzed using the K-means clustering algorithm [28]. Each individual’s standardized TyG-AIP values were used to group participants with similar change trends into clusters. The optimal number of clusters (K = 3) was determined by evaluating the elbow method, the average silhouette coefficient, and the clinical interpretability of cluster solutions (K tested from 2 to 5). Consequently, participants were classified into three subgroups representing distinct TyG-AIP change patterns (Fig. 2).

The associations between TyG-AIP change clusters and incident stroke were evaluated using multivariable Cox proportional hazards regression models. Three hierarchical models were constructed:

- Model 1: Adjusted for age and sex.
- Model 2: Further adjusted for sociodemographic factors (age, sex, and education).
- Model 3: Additionally adjusted for behavioral and clinical covariates, including smoking, drinking, BMI, and comorbidities, based on Model 2.
- Results are presented as hazard ratios (HRs) and 95% confidence intervals (CIs).

To explore the combined impact of TyG-AIP and hypertension, a composite exposure variable was generated by cross-classifying TyG-AIP change patterns with baseline hypertension status (yes/no). The joint association of this variable with stroke risk was assessed using Cox models. All analyses were conducted using R software (version 4.5.1), and a two-tailed *P* < 0.05 was considered statistically significant.

To assess the association between baseline TyG-AIP levels and stroke risk using a conventional approach, participants were also stratified into tertiles (T1-T3) and quartiles (Q1-Q4) based on their 2012 TyG-AIP value. Hazard ratios were calculated using Cox models with the lowest group as reference. The internal validity of the trajectory clusters was assessed using the silhouette coefficient, with values closer to 1 indicating better-defined clusters.

### Statistical analysis

All statistical analyses were conducted using R (version 4.5.1) and Python (version 3.11). To address missing data and reduce potential bias, Multiple Imputation by Chained Equations (MICE) was performed in Python (version 3.12.4). This method iteratively constructs regression models for each variable with missing values using information from other variables to generate multiple complete datasets. Outliers exceeding 1.5 times the interquartile range (IQR) for variables such as height, weight, and waist circumference were removed before imputation to avoid bias. A two-sided P value < 0.05 was considered statistically significant.

#### Descriptive and comparative analyses

Participants were categorized into four groups based on their TyG-AIP level (above or below the median) and hypertension status (yes or no). Continuous variables were examined for normality using the Shapiro–Wilk test. Normally distributed variables are presented as mean ± standard deviation (SD) and compared using one-way ANOVA. Categorical variables are expressed as frequencies (percentages) and compared using the χ^2^ test. Baseline characteristics were further summarized according to the occurrence of the endpoint event.

#### Incidence and stratified analyses

Cumulative incidence rates of stroke were calculated as the number of new stroke cases divided by the total number of participants at baseline, expressed per 1,000 persons. Stratified analyses were performed by age, sex, hypertension, smoking, and drinking status to evaluate potential effect modification. TyG-AIP was also treated as a continuous variable to assess robustness. Subgroup analyses were further conducted across categories of hypertension, age, sex, BMI, smoking, and drinking.

#### Cox proportional hazards models

Associations between TyG-AIP (alone and combined with hypertension) and incident stroke were assessed using Cox proportional hazards regression. The reference group comprised participants with both low TyG-AIP and no hypertension. Three hierarchical models were constructed:

- Model 1: Adjusted for age and sex.
- Model 2: Further adjusted for smoking status, drinking status, BMI, marital status, education level, and household registration.
- Model 3: Additionally adjusted for cancer, heart disease, dyslipidemia, diabetes, anti-diabetic medication, cardiac medication, LDL-C, total cholesterol, and glycated hemoglobin (HbA1c).

Results are expressed as HRs and 95% CIs. The proportional hazards assumption for key exposure variables (TyG, AIP, TyG-AIP, and TyG-AIP + hypertension) was verified using Schoenfeld residuals, and no violations were observed (all *P* > 0.05).

#### Nonlinear association and predictive analyses

To assess the dose–response relationship between TyG, AIP, and TyG-AIP and the risk of stroke, restricted cubic spline (RCS) models were fitted, adjusting for covariates under different glycemic status categories.

The predictive value of TyG-AIP and hypertension for stroke was evaluated and compared using Cox proportional hazards models and time-dependent receiver operating characteristic (ROC) curve analysis. To test our hypothesis that combining metabolic and hemodynamic risk factors would improve stroke risk prediction, we adopted a model comparison approach:

- Baseline models: Separate univariate Cox models were built with TyG-AIP (as a continuous variable) and hypertension (as a binary variable) as independent predictors.
- Combined model: A multivariate Cox model incorporating both TyG-AIP and hypertension as covariates was then constructed.
- Performance comparison: The discriminatory ability of each model was assessed and compared using the area under the ROC curve (AUC). The statistical significance of differences in AUC between the combined model and each single-factor model was evaluated to quantify the incremental predictive value gained by integrating both risk factors.

To assess the discriminative performance of TyG-AIP for stroke prediction, we performed ROC curve analyses. We calculated the area under the curve (AUC) with 95% confidence intervals for four predictors: hypertension, TyG, AIP, and TyG-AIP. The optimal cutoff point was determined using the Youden index, with corresponding sensitivity, specificity, positive predictive value (PPV), and negative predictive value (NPV) calculated. Statistical comparisons between AUCs were performed using the DeLong test. These analyses were conducted separately for the total population and for PDM and NDM subgroups.

To quantify the improvement in individual risk classification upon adding TyG-AIP, we calculated the continuous NRI. We compared a conventional model (adjusted for age, sex, and hypertension) with an enhanced model that additionally included TyG-AIP. The predicted absolute risks of stroke at the median follow-up time were derived from each Cox model. The NRI and its 95% confidence interval were estimated using the nricens package in R (version 4.5.1) with 1000 bootstrap resamples. This analysis was performed in the overall cohort and within the PDM and NDM subgroups.

To ensure robustness, results were revalidated using data from the 2015 survey. Participants with incomplete follow-up data were excluded to minimize attrition bias. All analyses were repeated in the complete-case cohort to confirm the consistency of the associations.

## Results

### Baseline characteristics of the 5786 included participants

Tables 1 and 2 summarize the baseline characteristics of the 5786 participants, stratified by the combination of TyG-AIP index and hypertension status, and by stroke occurrence, respectively.

**Table 1:** Baseline characteristics of the study participants stratified by TyG-AIP and hypertension status

Characters	Level	Overall	TyG_AIP ≤ median and no hypertension	TyG_AIP > median and no hypertension	TyG_AIP ≤ median and hypertension	TyG_AIP > median and hypertension	P
Number		5786	2132	1916	761	977	
Age, years (mean (SD))		59.3(9.4)	58.4(9.2)	57.8(8.6)	62.9(10.2)	61.4(9.6)	< 0.001
Age, years (%)	> 60	2349(40.6)	773(36.3)	662(34.6)	421(55.3)	493(50.5)	< 0.001
	45–60	3437(59.4)	1359(63.7)	1254(65.4)	340(44.7)	484(49.5)	
Sex (%)	Female	3124(54.0)	1104(51.8)	1081(56.4)	382(50.2)	557(57.0)	0.001
	Male	2662(46.0)	1028(48.2)	835(43.6)	379(49.8)	420(43.0)	
Education (%)	Below high school	5153(89.1)	1906(89.4)	1670(87.2)	707(92.9)	870(89.0)	< 0.001
	High school or above	633(10.9)	226(10.6)	246(12.8)	54(7.1)	107(11.0)	
Marital_status (%)	Married or partnered	5077(87.7)	1912(89.7)	1722(89.9)	611(80.3)	832(85.2)	< 0.001
	Separated	709(12.3)	220(10.3)	194(10.1)	150(19.7)	145(14.8)	
Hukou (%)	Rural	4736(81.9)	1795(84.2)	1527(79.7)	646(84.9)	768(78.6)	< 0.001
	Urban	1050(18.1)	337(15.8)	389(20.3)	115(15.1)	209(21.4)	
BMI, kg/m^2^ (mean (SD))		24.4 (33.9)	23.9 (54.6)	24.8 (11.3)	23.4 (4.8)	25.5 (5.0)	0.517
Smoking (%)	No	3507(60.6)	1284(60.2)	1172(61.2)	439(57.7)	612(62.6)	0.188
	Yes	2279(39.4)	848(39.8)	744(38.8)	322(42.3)	365(37.4)	
Drinking (%)	No	3891(67.2)	1384(64.9)	1326(69.2)	488(64.1)	693(70.9)	< 0.001
	Yes	1895(32.8)	748(35.1)	590(30.8)	273(35.9)	284(29.1)	
Heart_problem (%)	No	5028(86.9)	1925(90.3)	1633(85.2)	661(86.9)	809(82.8)	< 0.001
	Yes	758(13.1)	207(9.7)	283(14.8)	100(13.1)	168(17.2)	
Meds_heart_problems (%)	No	5284(91.3)	1985(93.1)	1737(90.7)	684(89.9)	878(89.9)	0.003
	Yes	502(8.7)	147(6.9)	179(9.3)	77(10.1)	99(10.1)	
Cancer (%)	No	5739(99.2)	2114(99.2)	1901(99.2)	757(99.5)	967(99.0)	0.714
	Yes	47(0.8)	18(0.8)	15(0.8)	4(0.5)	10(1.0)	
Meds_cancer (%)	No	5754(99.4)	2117(99.3)	1904(99.4)	761(100.0)	972(99.5)	0.149
	Yes	32(0.6)	15(0.7)	12(0.6)	0(0.0)	5(0.5)	
HBP (%)	No	4048(70.0)	2132(100.0)	1916(100.0)	0(0.0)	0(0.0)	< 0.001
	Yes	1738(30.0)	0(0.0)	0(0.0)	761(100.0)	977(100.0)	
Meds_HBP (%)	No	4571(79.0)	1958(91.8)	1572(82.0)	508(66.8)	533(54.6)	< 0.001
	Yes	1215(21.0)	174(8.2)	344(18.0)	253(33.2)	444(45.4)	
Meds_diabetes (%)	No	5523(95.5)	2069(97.0)	1812(94.6)	739(97.1)	903(92.4)	< 0.001
	Yes	263(4.5)	63(3.0)	104(5.4)	22(2.9)	74(7.6)	
Diabetes_status (%)	No	2296(39.7)	1047(49.1)	678(35.4)	322(42.3)	249(25.5)	< 0.001
	Pre-diabetes	2653(45.9)	906(42.5)	906(47.3)	357(46.9)	484(49.5)	
	Diabtes	837(14.5)	179(8.4)	332(17.3)	82(10.8)	244(25.0)	
Dyslipidemia (%)	No	5164(89.2)	2006(94.1)	1675(87.4)	693(91.1)	790(80.9)	< 0.001
	Yes	622(10.8)	126(5.9)	241(12.6)	68(8.9)	187(19.1)	
Meds_dyslipidemia (%)	No	5451(94.2)	2073(97.2)	1790(93.4)	718(94.3)	870(89.0)	< 0.001
	Yes	335(5.8)	59(2.8)	126(6.6)	43(5.7)	107(11.0)	
SBP, mm Hg (mean (SD))		130.0(21.2)	118.1(11.8)	120.1(11.4)	155.2(15.4)	155.7(16.1)	< 0.001
DBP, mm Hg (mean (SD))		75.4(12.0)	69.8(8.9)	71.6(8.7)	85.2(10.8)	87.2(11.1)	< 0.001
FPG, mmol/L (mean (SD))		6.2(2.0)	5.8(1.4)	6.4(2.4)	5.9(1.4)	6.8(2.6)	< 0.001
HbA1c, % (mean (SD))		5.3(0.8)	5.2(0.7)	5.4(0.9)	5.2(0.6)	5.5(1.1)	< 0.001
HDL-C, mmol/L (mean (SD))		51.0(15.3)	60.0(14.0)	41.6(9.7)	61.0(14.5)	41.9(10.1)	< 0.001
LDL-C, mmol/L (mean (SD))		117.9(35.8)	116.0(32.2)	117.7(37.9)	118.7(32.6)	122.0(41.0)	< 0.001
TC, mmol/L (mean (SD))		194.6(39.3)	188.2(36.3)	196.9(40.0)	192.9(36.7)	205.4(43.4)	< 0.001
TG, mmol/L (mean (SD))		134.0(108.4)	76.7(22.2)	184.5(115.7)	79.3(22.6)	202.6(150.7)	< 0.001
TyG index (mean (SD))		8.7(0.7)	8.2(0.4)	9.1(0.5)	8.3(0.3)	9.2(0.6)	< 0.001
AIP (mean (SD))		0.4(0.3)	0.1(0.2)	0.6(0.2)	0.1(0.2)	0.6(0.3)	< 0.001
TyG-AIP (mean (SD))		3.3(3.3)	0.9(1.4)	5.7(2.6)	0.9(1.4)	6.0(3.1)	< 0.001

TyG, triglyceride-glucose index; AIP, atherogenic index of plasma; SBP, systolic blood pressure; DBP, diastolic blood pressure; FBG, fasting blood glucose; HbA1c, glycated hemoglobin; HDL, high-density lipoprotein; LDL, low-density lipoprotein; TC, total cholesterol; TG, triglycerides; HBP, high blood pressure

Data are presented as mean (standard deviation) for continuous variables and number (percentage) for categorical variables. Participants were divided into four groups based on their TyG-AIP level (using the study population median as the cut-off point) and hypertension status. The P-values were derived from one-way ANOVA for continuous variables and the Chi-square test for categorical variables to test differences across the four groups

**Table 2:** Baseline characteristics of the study participants stratified by incident stroke status during follow-up

Characters	Level	Overall	Non-stroke	Stroke	P
Number		5786	5326	460	
Age, years (mean (SD))		59.3(9.4)	59.2(9.4)	60.9(8.4)	< 0.001
Age, years (%)	> 60	2349(40.6)	2125(39.9)	224(48.7)	< 0.001
	45–60	3437(59.4)	3201(60.1)	236(51.3)	
Sex(%)	Female	3124(54.0)	2885(54.2)	239(52.0)	0.387
	Male	2662(46.0)	2441(45.8)	221(48.0)	
Education (%)	Below high school	5153(89.1)	4738(89.0)	415(90.2)	0.453
	High school or above	633(10.9)	588(11.0)	45(9.8)	
Marital_status (%)	Married or partnered	5077(87.7)	4691(88.1)	386(83.9)	0.011
	Separated	709(12.3)	635(11.9)	74(16.1)	
Hukou (%)	Rural	4736(81.9)	4364(81.9)	372(80.9)	0.612
	Urban	1050(18.1)	962(18.1)	88(19.1)	
BMI, kg/m^2^ (mean (SD))	Abnormal BMI	2294(39.6)	2078(39.0)	216(47.0)	0.001
	Normal BMI	3492(60.4)	3248(61.0)	244(53.0)	
Smoking (%)	No	3507(60.6)	3244(60.9)	263(57.2)	0.128
	Yes	2279(39.4)	2082(39.1)	197(42.8)	
Drinking (%)	No	3891(67.2)	3588(67.4)	303(65.9)	0.545
	Yes	1895(32.8)	1738(32.6)	157(34.1)	
Heart_problem (%)	No	5028(86.9)	4664(87.6)	364(79.1)	< 0.001
	Yes	758(13.1)	662(12.4)	96(20.9)	
Meds_heart_problems (%)	No	5284(91.3)	4882(91.7)	402(87.4)	0.002
	Yes	502(8.7)	444(8.3)	58(12.6)	
Meds_stroke (%)	No	5768(99.7)	5324(100.0)	444(96.5)	< 0.001
	Yes	18(0.3)	2(0.0)	16(3.5)	
Cancre (%)	No	5739(99.2)	5282(99.2)	457(99.3)	0.898
	Yes	47(0.8)	44(0.8)	3(0.7)	
Meds_cancer (%)	No	5754(99.4)	5294(99.4)	460(100.0)	0.18
	Yes	32(0.6)	32(0.6)	0(0.0)	
HBP (%)	No	4048(70.0)	3786(71.1)	262(57.0)	< 0.001
	Yes	1738(30.0)	1540(28.9)	198(43.0)	
HTN (%)	No	4078(70.5)	3810(71.5)	268(58.3)	< 0.001
	Yes	1708(29.5)	1516(28.5)	192(41.7)	
Meds_HBP (%)	No	4571(79.0)	4306(80.8)	265(57.6)	< 0.001
	Yes	1215(21.0)	1020(19.2)	195(42.4)	
Meds_diabetes (%)	No	5523(95.5)	5099(95.7)	424(92.2)	0.001
	Yes	263(4.5)	227(4.3)	36(7.8)	
Diabetes_status (%)	No	2296(39.7)	2145(40.3)	151(32.8)	0.002
	Pre-diabetes	2653(45.9)	2429(45.6)	224(48.7)	
	Diabtes	837(14.5)	752(14.1)	85(18.5)	
Dyslipidemia (%)	No	5164(89.2)	4807(90.3)	357(77.6)	< 0.001
	Yes	622(10.8)	519(9.7)	103(22.4)	
Meds_dyslipidemia (%)	No	5451(94.2)	5043(94.7)	408(88.7)	< 0.001
	Yes	335(5.8)	283(5.3)	52(11.3)	
SBP, mm Hg (mean (SD))		130.0(21.2)	129.4(20.9)	137.2(22.9)	< 0.001
DBP, mm Hg (mean (SD))		75.4(12.0)	75.0(11.9)	79.0(12.4)	< 0.001
FPG, mmol/L (mean (SD))		6.2(2.0)	6.2(2.0)	6.5(2.2)	0.001
HbA1c, % (mean (SD))		5.3(0.8)	5.3(0.8)	5.4(0.9)	0.002
HDL-C, mmol/L (mean (SD))		51.0(15.3)	51.3(15.3)	47.2(14.2)	< 0.001
LDL-C, mmol/L (mean (SD))		117.9(35.8)	117.8(35.7)	119.9(37.0)	0.223
TC, mmol/L (mean (SD))		194.6(39.3)	194.3(39.3)	197.9(39.2)	0.059
TG, mmol/L (mean (SD))		134.0(108.4)	132.4(108.7)	152.4(103.5)	< 0.001
TyG index (mean (SD))		8.7(0.7)	8.7(0.7)	8.9(0.7)	< 0.001
AIP (mean (SD))		0.4(0.3)	0.4(0.3)	0.5(0.3)	< 0.001
TyG_AIP (mean (SD))		3.3(3.3)	3.3(3.3)	4.3(3.4)	< 0.001

SBP, systolic blood pressure; DBP, diastolic blood pressure; FBG, fasting blood glucose; HbA1c, glycated hemoglobin; HDL, high-density lipoprotein; LDL, low-density lipoprotein; TC, total cholesterol; TG, triglycerides; HBP, high blood pressure (self-reported); HTN, hypertension (measured); TyG, triglyceride-glucose index; AIP, atherogenic index of plasma

Data are presented as mean (standard deviation) for continuous variables and number (percentage) for categorical variables. The *P*-values were derived from Student’s t-test (for continuous variables) and the Chi-square test (for categorical variables) to test differences between the non-stroke and stroke groups

The mean age of all participants was 59.3 (SD 9.4), with 59.4% aged between 45 and 60 years. Females accounted for a higher proportion than males (54% vs. 46%). Most participants had an education level below high school (89.1%), were married or partnered (87.7%), and had rural household registration (81.9%). The proportions of non-smokers and non-drinkers both exceeded 50%.

Based on the median TyG-AIP and hypertension status, participants were categorized into four groups: TyG-AIP ≤ median and no hypertension (reference group, n = 2,132), TyG-AIP > median and no hypertension (n = 1,916), TyG-AIP ≤ median and hypertension (n = 761), and TyG-AIP > median and hypertension (n = 977). Except for BMI, smoking status, cancer history, and use of anticancer medications, significant differences were observed across the four groups for all other baseline variables (all *P* < 0.05). With increasing TyG-AIP and hypertension burden, age, BMI, blood pressure, glucose-related indicators (FBG, HbA1c), LDL-C, and total cholesterol exhibited upward trends, while HDL-C and triglycerides showed fluctuating changes. The TyG-AIP > median with hypertension group demonstrated the most adverse metabolic and atherogenic profile, indicating it may carry the highest stroke risk burden.

Compared with the non-stroke group, participants in the stroke group were older and had higher rates of abnormal BMI, heart disease, hypertension, diabetes, and dyslipidemia. Metabolic abnormalities were more pronounced in the stroke group, with significantly higher levels of the TyG index (8.9 vs. 8.7), AIP (0.5 vs. 0.4), and TyG-AIP (4.3 vs. 3.3). No significant differences were found for gender, education level, marital status, household registration, smoking, alcohol consumption, cancer history, anticancer medication use, LDL-C, or total cholesterol.

### The highest cumulative incidence of stroke was observed in the presence of both high TyG-AIP and hypertension

**Fig. 3:**
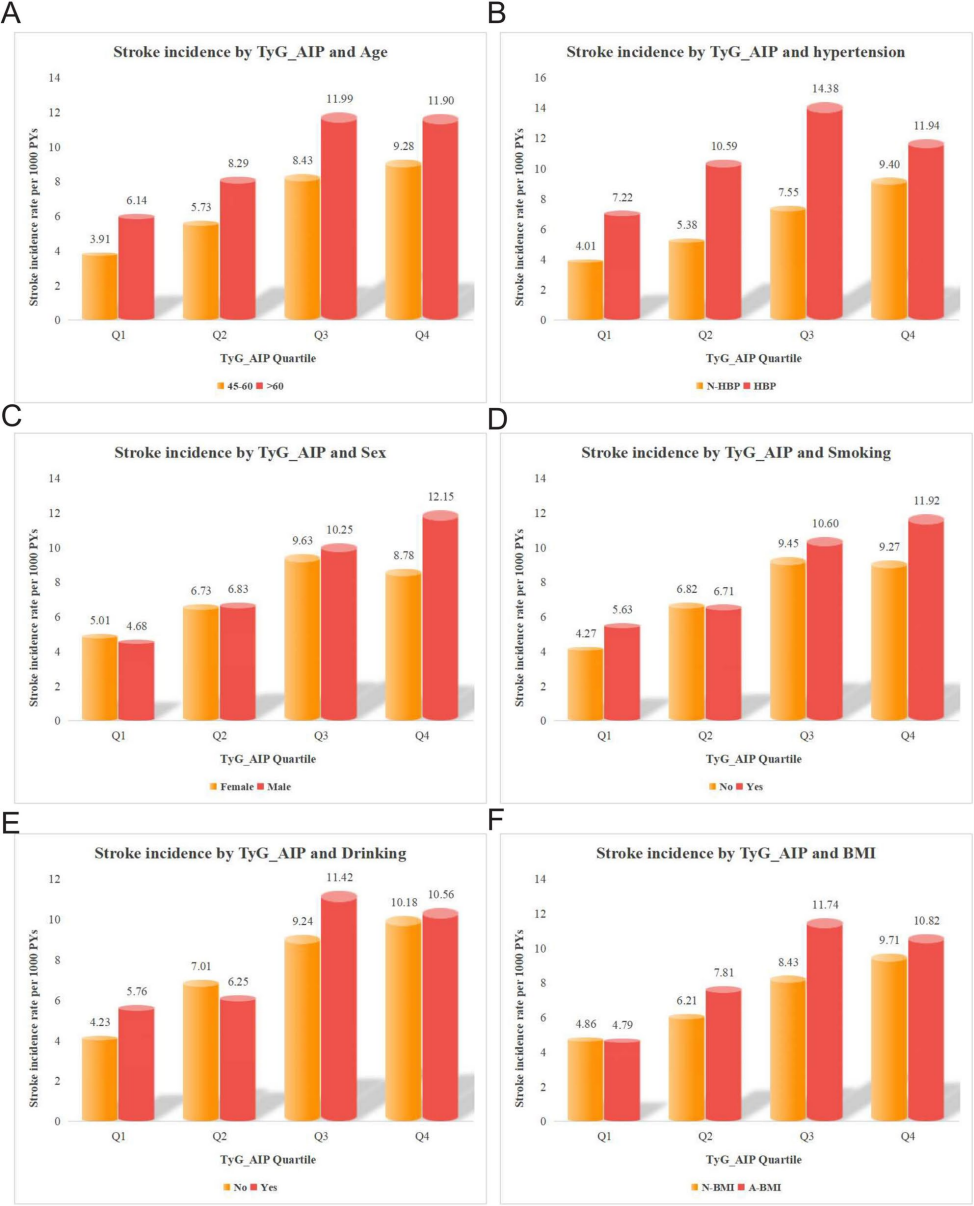
Results of TyG-AIP and Cumulative Incidence of Stroke Stratified by Sex, Age, Hypertension, Smoking, Alcohol Consumption, and BMI

As shown in Fig. 3, a total of 460 incident stroke cases were recorded during the follow-up period. Analysis of specific subgroups revealed that the cumulative incidence of stroke was lowest (4.84%) in the group with low TyG-AIP and no hypertension, and gradually increased with rising TyG-AIP levels, reaching the highest rate (10.30%) in the high TyG-AIP group (Table 3).

Further subgroup analyses identified distinct risk patterns across populations. Among males, the cumulative incidence of stroke under high TyG-AIP status (12.15%) was higher than that among females (8.78%). Age stratification showed that participants aged ≥ 60 years had a higher incidence rate (11.20%) compared to those aged 45–59 years (9.20%), a trend independent of TyG-AIP status. Smokers also consistently exhibited a higher cumulative incidence (11.92%) than non-smokers (9.26%). However, for BMI and alcohol consumption status, the highest cumulative incidence was observed when TyG-AIP was in the third quartile (Q3), with rates reaching 11.74% in individuals with abnormal BMI and 11.42% in drinkers, respectively.

Notably, when stratified by hypertension status, the association between TyG-AIP and cumulative stroke incidence was most pronounced among hypertensive individuals. In this subgroup, the cumulative incidence in the TyG-AIP Q3 group reached 14.38%, substantially exceeding that of any other subgroup.

### The metabolic-atherogenic risk index is significantly associated with stroke risk under dysglycemic conditions

**Fig. 4:**
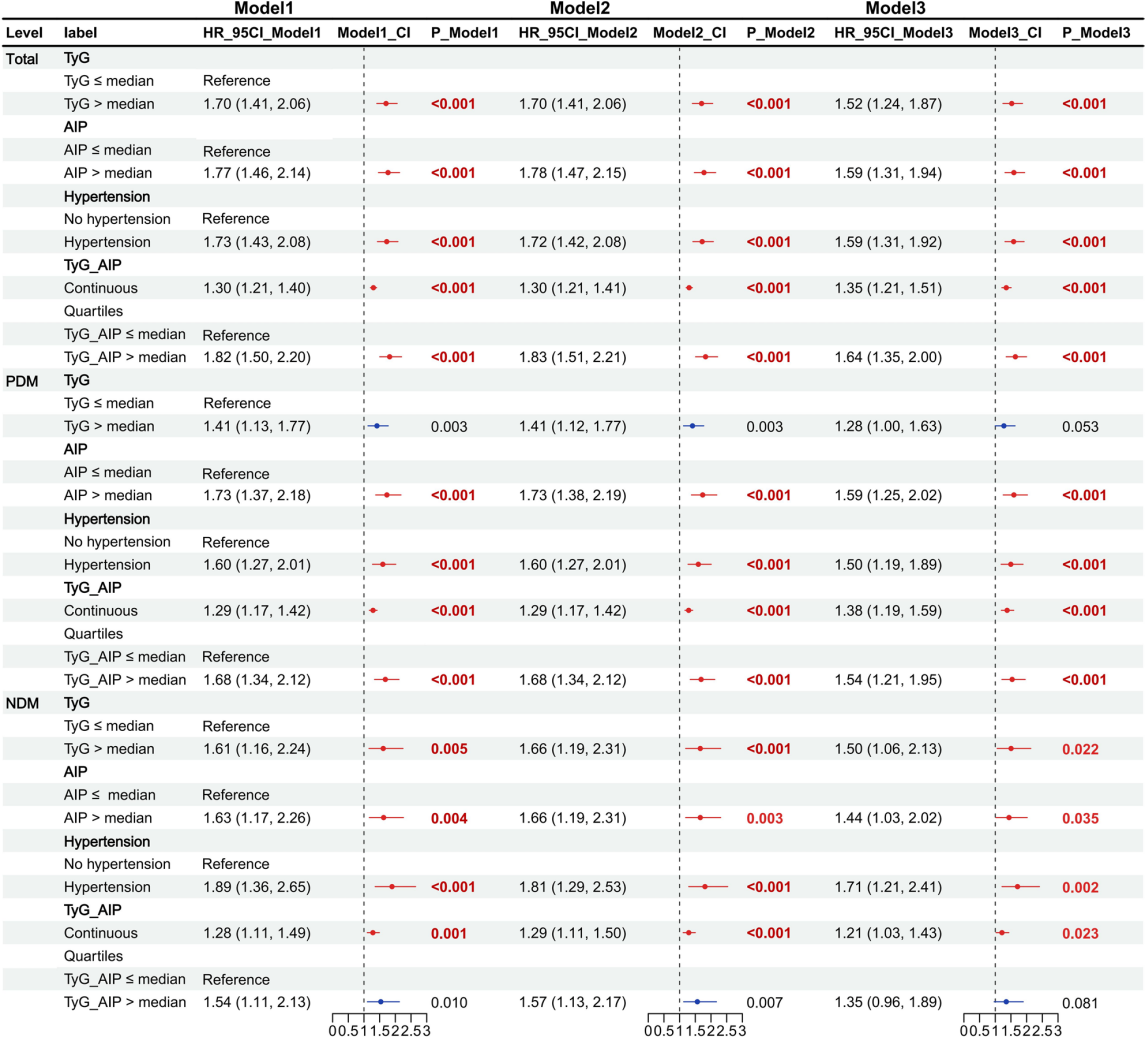
TyG, AIP and TyG-AIP as continuous and categorical variables with stroke risk in the total population, individuals with dysglycemia (PDM), and those with normoglycemia (NDM)

In the overall population, using groups with TyG and AIP ≤ median as the reference, we found that both TyG (HR = 1.59, 95% CI 1.31–1.94, *P* = 3.08E-06) and AIP (HR = 1.52, 95% CI 1.24–1.87, *P* = 5.01E-05) were significantly associated with stroke risk (Fig. 4). Using non-hypertension as the reference, hypertension was also significantly associated with stroke risk (HR = 1.59, 95% CI 1.31–1.92, *P* = 2.34E-06). Furthermore, TyG-AIP was significantly associated with stroke risk both as a continuous variable (HR = 1.35, 95% CI 1.21–1.51, *P* = 8.63E-08) and as a categorical variable (HR = 1.64, 95% CI 1.35–2.00, *P* = 8.02E-07).

In the dysglycemia population, using ≤ median or non-hypertension as the reference groups, we similarly found that AIP (HR = 1.59, 95% CI 1.25–2.02, *P* = 1.36E-04), hypertension (HR = 1.50, 95% CI 1.19–1.89, *P* = 6.66E-04), and TyG-AIP (HR = 1.54, 95% CI 1.21–1.95, *P* = 4.17E-04) increased the risk of stroke. However, the association between TyG and stroke risk was not confirmed (HR = 1.28, 95% CI 1.00–1.63, *P* = 5.35E-02).

In the normoglycemia population, using ≤ median or non-hypertension as the reference groups, we found that TyG (HR = 1.50, 95% CI 1.06–2.13, *P* = 2.22E-02), AIP (HR = 1.44, 95% CI 1.03–2.02, *P* = 3.53E-02), and hypertension (HR = 1.71, 95% CI 1.21–2.41, *P* = 2.17E-03) were all associated with stroke. However, the association between TyG-AIP (HR = 1.35, 95% CI 0.96–1.89, *P* = 8.06E-02) and stroke risk was not found to be statistically significant.

### Nonlinear association of TyG-AIP with stroke risk in the dysglycemia population

**Fig. 5:**
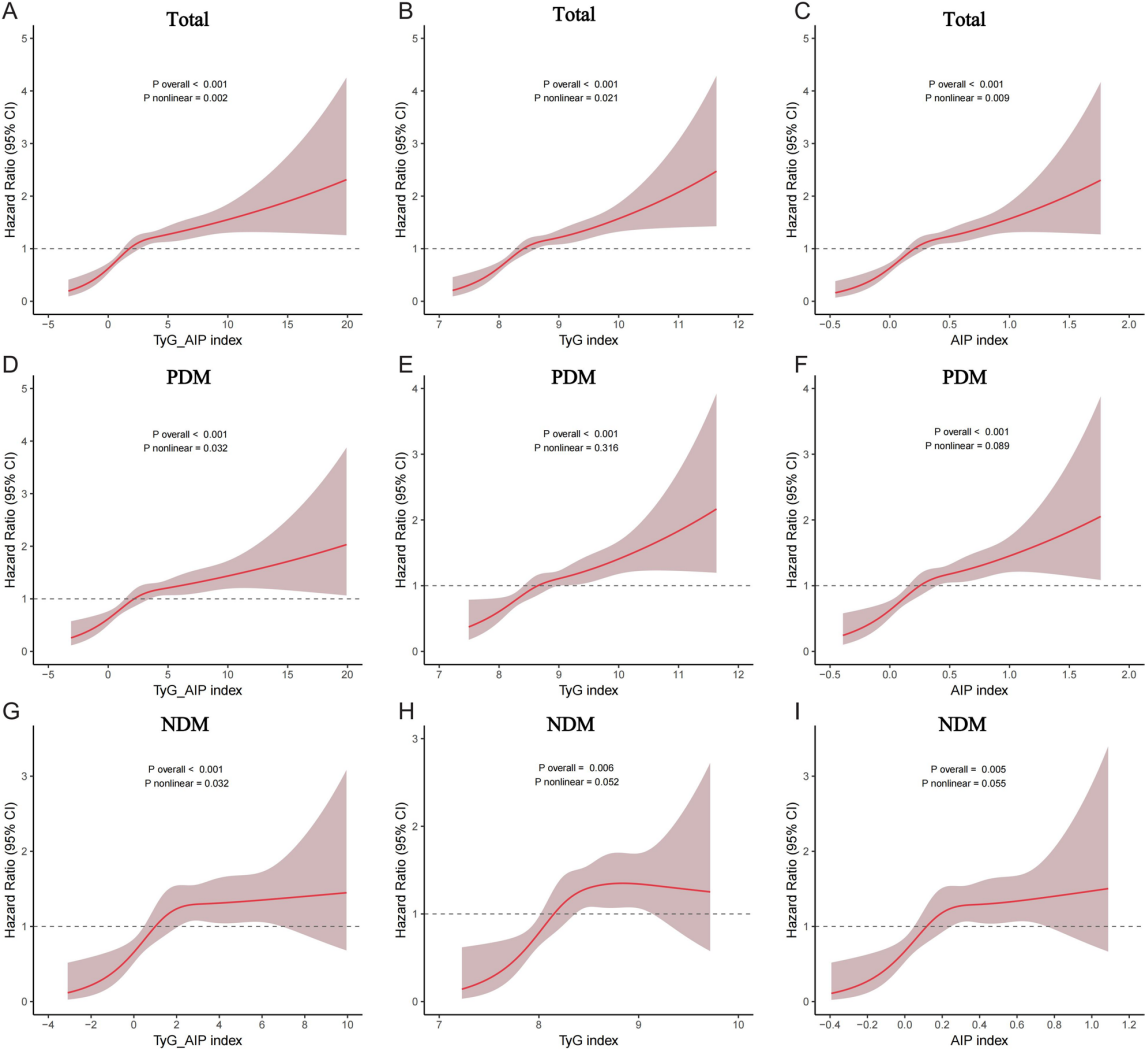
The dose–response relationship between TyG, AIP, TyG-AIP and stroke risk in the total population, individuals with dysglycemia (PDM), and those with normoglycemia (NDM). Notably, the RCS curves for TyG–AIP, TyG, and AIP exhibit highly similar shapes. This reflects that the TyG–AIP index successfully integrates the core metabolic risk signal related to stroke shared by its components. The incremental value of this composite index lies primarily in its clinical convenience as a unified metric and its superior predictive performance in multivariable-adjusted models (see Table 2 and Supplementary tables)

As shown in the RCS analysis (Fig. 5), TyG, AIP, and TyG-AIP all demonstrated significant overall associations with stroke risk (P_overall_ < 0.0001). Further analysis indicated nonlinear trends (P_nonlinear_ = 0.021, 0.009, and 0.002, respectively), with risk increasing as index values rose. In the PDM population, TyG-AIP showed a significant nonlinear pattern (P_nonlinear_ = 0.032), whereas in the NDM population the relationship appeared linear (P_nonlinear_ > 0.05), suggesting a possible threshold effect.

**Table 3:** Threshold effect analysis of TyG, AIP, and TyG-AIP on stroke risk using piecewise linear regression in the total and stratified populations

Populations	Index	Model 1: Standard linear regression (HR, 95%CI, P)	Inflection point	< Inflection (HR, 95%CI, P)	≥ Inflection (HR, 95%CI, P)	P for likelihood ratio test
Total	TyG	1.519(1.332–1.728), < 0.001	7.987	30.43 (4.13–382.04), 0.003	1.40(1.21–1.61), < 0.001	< 0.001
	AIP	2.397(1.835–3.122), < 0.001	0.357	7.295(3.453–16.05), < 0.001	1.449(0.936–2.197),0.088	< 0.001
	TyG_AIP	1.084 (1.057–1.112), < 0.001	3.128	1.26 (1.157–1.377), < 0.001	1.034 (0.994–1.073),0.083	< 0.001
PDM	TyG_AIP	1.071(1.041–1.102), < 0.001	3.386	1.232(1.115–1.37), < 0.001	1.029(0.985–1.071),0.189	0.004
NDM	TyG_AIP	1.108(1.04–1.178),0.001	1.343	1.666(1.239–2.352), 0.002	1.023(0.936–1.114),0.603	0.004

HR, hazard ratio; CI, confidence interval; TyG, triglyceride-glucose index; AIP, atherogenic index of plasma; PDM, population with dysglycemia; NDM, population with normoglycemia

This table presents the results of piecewise linear regression models, which were used to identify potential inflection points (thresholds) in the relationship between indices and stroke risk. Model 1 represents a standard Cox proportional hazards model treating the index as a continuous linear variable. The inflection point is the value at which the linear relationship significantly changes, as detected by the likelihood ratio test (*P* < 0.05 indicates the two-piece model fits significantly better than a simple linear model). HRs are presented separately for values below and above the inflection point

Piecewise linear regression identified inflection points at TyG = 7.987, AIP = 0.357, and TyG-AIP = 3.128. Below these points, HRs were 30.43 (95% CI 4.13–382.04), 7.295 (95% CI 3.453–16.05), and 1.26 (95% CI 1.157–1.377), respectively; above the inflection points, HRs decreased substantially. Similar nonlinear trends were observed in both PDM and NDM subgroups, indicating threshold effects between these indices and stroke risk.

As shown in Fig. 5, TyG–AIP, TyG, and AIP all demonstrated significant nonlinear dose–response relationships with stroke risk (P for nonlinearity < 0.05 for all). It is noteworthy that the curves for the three indices are highly congruent. This consistency validates, from different measurement perspectives, the robust association between the core metabolic risk (encompassing insulin resistance and atherogenic dyslipidemia) and stroke. However, univariate RCS analysis primarily reveals risk trends; the practical utility of TyG–AIP as a composite metric and its predictive superiority over individual components are more critically demonstrated in subsequent multivariable-adjusted models and incremental value analyses (see Supplemental Table 11).

### Risk stratification by combined TyG–AIP and hypertension categories is independent of glycemic status

Formal testing for a multiplicative interaction between continuous TyG–AIP and hypertension on stroke risk was not statistically significant (*P* > 0.05). Nevertheless, to address our primary aim of identifying high-risk clinical phenotypes, we performed a joint stratification analysis.

**Fig. 6:**
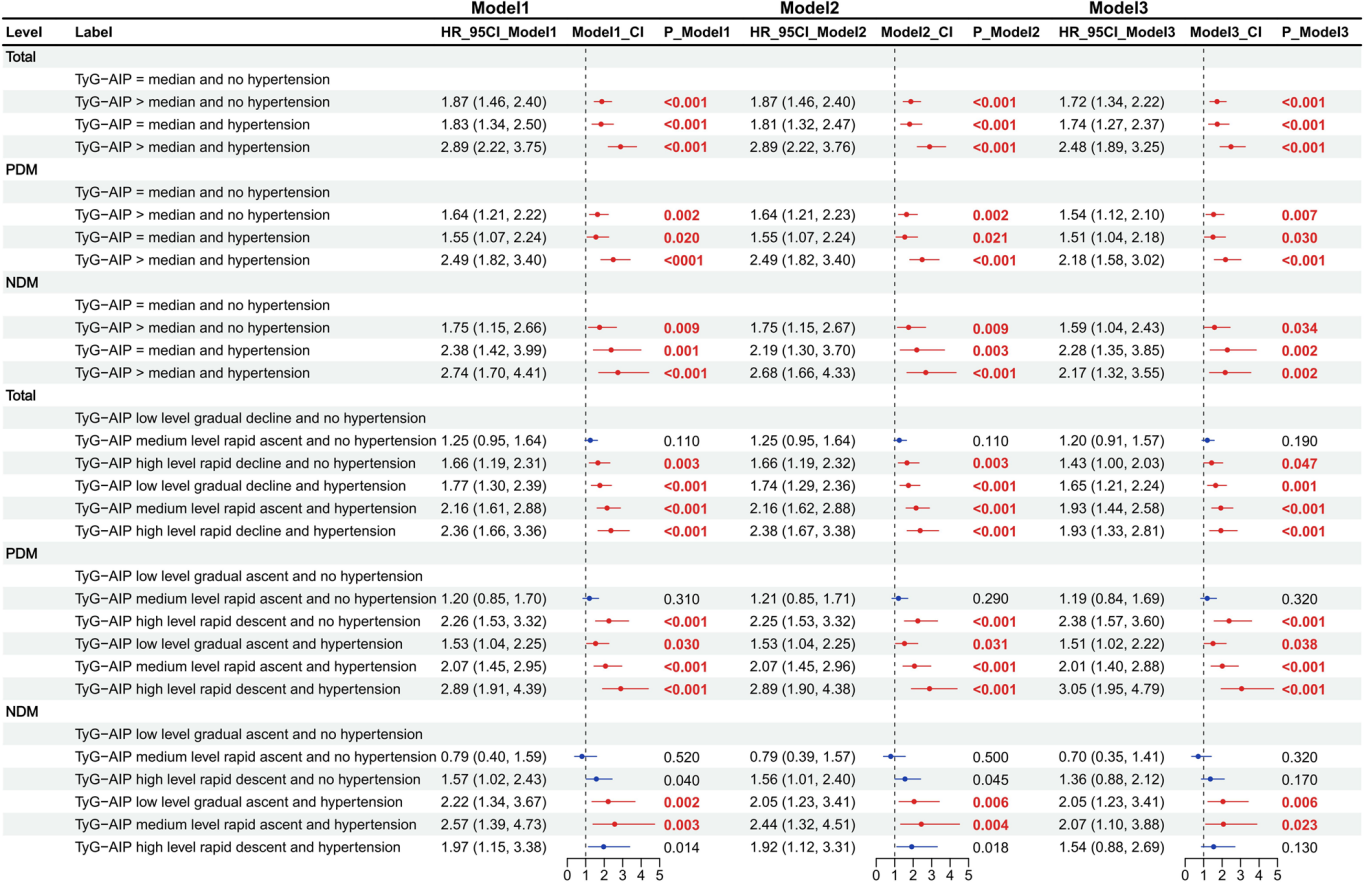
TyG-AIP index + hypertension and TyG-AIP index trajectory + hypertension with stroke risk in the total population, individuals with dysglycemia (PDM), and those with normoglycemia (NDM). The figure displays all groups relative to a common reference (Q1). For direct comparisons assessing the specific contributions of hypertension and elevated TyG-AIP within strata (e.g., Q4 vs. Q2; Q4 vs. Q3), please refer to Supplementary Tables 13, 14

In the overall population, using the “TyG–AIP ≤ median and no hypertension” group as reference, stroke risk increased progressively across the combined categories. The hazard was elevated in groups with a single risk factor—“TyG–AIP > median and no hypertension” (HR = 1.72, 95% CI 1.34–2.22) and “TyG–AIP ≤ median and hypertension” (HR = 1.74, 95% CI 1.27–2.37). Critically, individuals in the “TyG–AIP > median and hypertension” group—representing the dual metabolic-hemodynamic burden—exhibited the highest risk, with an HR of 2.48 (95% CI 1.89–3.25). This risk estimate was substantially greater than that observed in either of the single-exposure groups. A significant trend across the four categories was observed (P for trend < 0.001). Kaplan–Meier curves (Fig. 6) visually corroborated this pronounced gradient in cumulative event rates.

Similar risk patterns were observed in both the PDM and NDM subgroups, indicating that the adverse impact of the combined TyG–AIP and hypertension burden on stroke risk is consistent and independent of baseline glycemic status.

To address the potential modifying effect of hypertension on the association between TyG-AIP trajectories and cardiovascular outcomes, we performed supplementary analyses including formal interaction tests and stratified comparisons (Supplementary Table 13). Although multiplicative interaction terms were not statistically significant in any population (all *P* > 0.05), stratified analyses revealed important patterns of effect modification.

In the total and non-diabetes populations, hypertension dramatically altered the risk landscape. Among non-hypertensive individuals, a high-risk TyG-AIP trajectory showed borderline significant increased risk (HR = 1.97, 95% CI 0.99–3.92, *P* = 0.055) compared to a low-risk trajectory. Strikingly, among hypertensive patients, neither medium- nor high-risk trajectories conferred significant additional risk beyond that of hypertension alone. This pattern suggests that in normoglycemic individuals, hypertension may dominate the risk profile, potentially masking additional contributions from unfavorable metabolic trajectories.

In contrast, among prediabetic individuals, both medium- and high-risk TyG-AIP trajectories remained significant risk factors even in the presence of hypertension (HR = 1.32, 95% CI 0.84–2.07 and HR = 1.77, 95% CI 1.11–2.82, respectively). Hypertension itself was consistently a powerful independent risk factor across all populations, particularly among those with low-risk TyG-AIP trajectories (HR = 3.38 in total/NDM populations, HR = 1.63 in PDM population).

### Associations of TyG-AIP trajectories and hypertension with stroke risk

Cox regression analyses demonstrated that TyG-AIP trajectory patterns were significantly associated with stroke risk (Table 4). Compared with the “low level gradual decline” trajectory, individuals following a “high level rapid decline” pattern had 36% higher risk (HR = 1.36, 95% CI 1.03–1.79; *P* = 0.030). In the PDM population, this association was particularly pronounced (HR = 2.26, 95% CI 1.62–3.15; *P* < 0.001).

**Table 4:** Association between longitudinal trajectories of TyG-AIP and the risk of incident stroke in total and stratified populations

Populations	Levels	Model	HR(95%CI)	P value
*Total*				
	TyG-AIP low level gradual decline	Reference		
	TyG-AIP medium level rapid ascent	Model 1	1.28(1.04–1.57)	0.020
	TyG-AIP medium level rapid ascent	Model 2	1.28(1.05–1.58)	0.017
	TyG-AIP medium level rapid ascent	Model 3	1.22(0.99–1.50)	0.063
	TyG-AIP high level rapid decline	Model 1	1.61(1.25–2.06)	** < 0.001**
	TyG-AIP high level rapid decline	Model 2	1.62(1.27–2.08)	** < 0.001**
	TyG-AIP high level rapid decline	Model 3	1.36(1.03–1.79)	**0.030**
*PDM*				
	TyG-AIP low level gradual ascent	Reference		
	TyG-AIP medium level rapid ascent	Model 1	1.30(1.00–1.68)	0.494
	TyG-AIP medium level rapid ascent	Model 2	1.30(1.00–1.69)	0.458
	TyG-AIP medium level rapid ascent	Model 3	1.28(0.99–1.67)	0.615
	TyG-AIP high level rapid descent	Model 1	2.17(1.62–2.91)	** < 0.001**
	TyG-AIP high level rapid descent	Model 2	2.17(1.61–2.91)	** < 0.001**
	TyG-AIP high level rapid descent	Model 3	2.26(1.62–3.15)	** < 0.001**
*NDM*				
	TyG-AIP low level gradual ascent	Reference		
	TyG-AIP high level rapid ascent	Model 1	1.05(0.66–1.67)	0.842
	TyG-AIP high level rapid ascent	Model 2	1.05(0.65–1.67)	0.852
	TyG-AIP high level rapid ascent	Model 3	0.91(0.57–1.46)	0.697
	TyG-AIP medium level rapid descent	Model 1	1.33(0.94–1.88)	0.110
	TyG-AIP medium level rapid descent	Model 2	1.34(0.94–1.90)	0.103
	TyG-AIP medium level rapid descent	Model 3	1.14(0.79–1.63)	0.490

Boldface numbers indicate that the corresponding hazard ratio (HR) isstatistically significant (i.e., its 95% confidence interval excludes 1.00, and the P-value < 0.05)

HR, hazard ratio; CI, confidence interval; TyG-AIP, combined triglyceride-glucose and atherogenic index of plasma; PDM, population with dysglycemia; NDM, population with normoglycemia

Distinct longitudinal trajectories of TyG-AIP from 2012 to 2015 were identified using K-means clustering analysis on standardized values. The optimal number of clusters was determined as three (K = 3) based on the elbow method, average silhouette coefficient, and clinical interpretability, corresponding to the patterns described in the “Levels” column (e.g., “low level gradual decline”). The cluster with the theoretically lowest risk pattern within each population was set as the reference group. Model 1 was adjusted for age and sex. Model 2 was further adjusted for sociodemographic factors (age, sex, and education). Model 3 was additionally adjusted for behavioral and clinical covariates (smoking, drinking, BMI, and comorbidities). *P*-values are reported in scientific notation

**Fig. 7:**
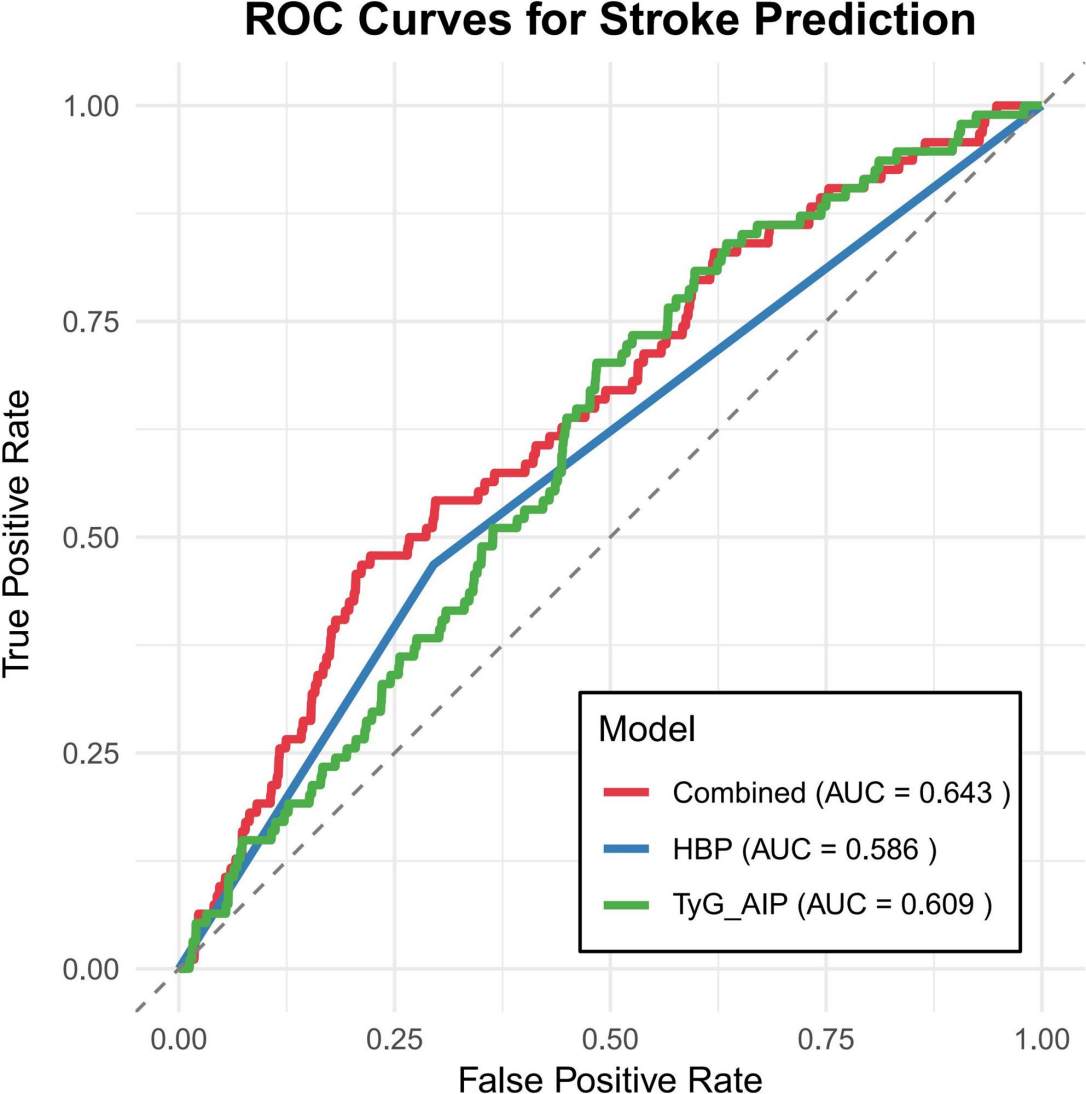
Superior predictive performance of a model combining TyG-AIP and hypertension for stroke risk. Receiver operating characteristic (ROC) curves compare three models: hypertension alone (blue), TyG-AIP index alone (green), and a combined model including both predictors (red). The area under the curve (AUC) for each model is shown in the legend. The combined model demonstrates the highest discriminatory ability, justifying the integration of both risk factors to improve prediction accuracy

When trajectory groups were combined with hypertension status (Fig. 7), joint associations emerged. In the overall population, participants with “high level rapid decline and hypertension” showed the highest risk (HR = 2.38, 95% CI 1.67–3.38;* P* < 0.001). Similar or stronger patterns were observed in PDM, while in NDM, associations were mainly driven by TyG-AIP trajectories themselves rather than hypertension.

In supplementary analyses, both tertile and quartile stratification of baseline TyG-AIP confirmed a strong, graded association with increased stroke risk across all populations (*P* for trend < 0.001; see Supplemental Table 12). K-means clustering on the longitudinal TyG-AIP data yielded three trajectory patterns. The average silhouette coefficient for this clustering was 0.479, indicating a fair level of structure within the continuum of metabolic phenotypes. Despite visual overlap in the 2D projection (Fig. 2A, D, G), these clusters exhibited significantly different stroke risks (Table 4).

### Predictive value of combined TyG-AIP and hypertension for stroke

We evaluated whether combining TyG-AIP with hypertension enhanced stroke prediction. As shown in Fig. 7, the model incorporating both risk factors demonstrated the highest discriminatory ability, with an AUC of 0.643. This performance was superior to that of the model containing only hypertension (AUC = 0.586) or only TyG-AIP (AUC = 0.609), indicating that the combination provides incremental predictive value.

The discriminative performance of TyG-AIP and comparator indicators is presented in Supplemental Table 13. In the total population, TyG-AIP showed an AUC of 0.590 (95% CI 0.565–0.616), which was not statistically different from TyG (AUC = 0.586, P = 0.447), AIP (AUC = 0.590, P = 0.287), or hypertension (AUC = 0.571, P = 0.258) by DeLong test. At the optimal cutoff of 2.795, TyG-AIP demonstrated 65.2% sensitivity and 50.0% specificity. Similar patterns were observed in PDM and NDM subgroups, with no statistically significant differences between TyG-AIP and comparator indicators in any population.

The NRI analysis demonstrated that the addition of TyG-AIP to the conventional risk factor model significantly improved the reclassification of individual stroke risk (Supplemental Tables 10, 11). In the overall population, the continuous NRI was 0.193 (95% CI 0.101–0.288). This improvement was consistently observed in both the dysglycemia subgroup (NRI = 0.162, 95% CI 0.045–0.280) and the normoglycemia subgroup (NRI = 0.190, 95% CI 0.030–0.346).

### Subgroup and interaction analyses

Subgroup analyses (Table 5, Supplementary Tables 1–9) demonstrated that “high TyG-AIP combined with hypertension” consistently represented the highest-risk combination across most subgroups. Interaction analyses identified education level as a significant effect modifier (*P*_interaction_ = 0.036), with particularly high risk observed among individuals with higher education (HR = 9.74). Borderline interaction was noted for gender (*P*_interaction_ = 0.127).

**Table 5:** Subgroup analysis of the association between the combined TyG-AIP and hypertension exposure and the risk of incident stroke

Variable	N	Percent	Levels	OR	HR_LL	HR_UL	OR(95%CI)	P value	P for interaction
Sex									0.127
Female	3124	54	TyG_AIP ≤ median and no hypertension	Reference					
			TyG_AIP > median and no hypertension	1.48	1.06	2.06	1.48(1.06–2.06)	0.022	
			TyG_AIP ≤ median and hypertension	1.44	0.92	2.25	1.44(0.92–2.25)	0.107	
			TyG_AIP > median and hypertension	2.34	1.65	3.32	2.34(1.65–3.32)	< 0.001	
Male	2662	46	TyG_AIP ≤ median and no hypertension	Reference					
			TyG_AIP > median and no hypertension	2.41	1.65	3.52	2.41(1.65–3.52)	< 0.001	
			TyG_AIP ≤ median and hypertension	2.66	1.72	4.13	2.66(1.72–4.13)	< 0.001	
			TyG_AIP > median and hypertension	4.01	2.7	5.94	4.01(2.10–5.94)	< 0.001	
Education									0.036
Below high school	5153	89.1	TyG_AIP ≤ median and no hypertension	Reference					
			TyG_AIP > median and no hypertension	1.67	1.29	2.16	1.67(1.29–2.16)	< 0.001	
			TyG_AIP ≤ median and hypertension	1.76	1.28	2.42	1.76(1.28–2.42)	0.001	
			TyG_AIP > median and hypertension	2.77	2.12	3.63	2.77(2.12–3.63)	< 0.001	
High school or above	633	10.9	TyG_AIP ≤ median and no hypertension	Reference					
			TyG_AIP > median and no hypertension	7.37	2.21	24.54	7.37(2.21–24.54)	0.001	
			TyG_AIP ≤ median and hypertension	8.69	2.17	34.76	8.69(2.17–34.76)	0.002	
			TyG_AIP > median and hypertension	9.74	2.78	34.19	9.74(2.78–34.19)	< 0.001	
Smoking									0.384
No	3507	60.6	TyG_AIP ≤ median and no hypertension	Reference					
			TyG_AIP > median and no hypertension	1.59	1.15	2.2	1.59(1.15–2.20)	0.005	
			TyG_AIP ≤ median and hypertension	1.55	1.02	2.37	1.55(1.02–2.37)	0.04	
			TyG_AIP > median and hypertension	2.68	1.92	3.74	2.68(1.92–3.74)	< 0.001	
Yes	2279	39.4	TyG_AIP ≤ median and no hypertension	Reference					
			TyG_AIP > median and no hypertension	2.28	1.54	3.39	2.28(1.54–3.39)	< 0.001	
			TyG_AIP ≤ median and hypertension	2.58	1.62	4.09	2.58(1.62–4.09)	< 0.001	
			TyG_AIP > median and hypertension	3.53	2.32	5.37	3.53(2.32–5.37)	< 0.001	
Drinking									0.653
No	3891	67.2	TyG_AIP ≤ median and no hypertension	Reference					
			TyG_AIP > median and no hypertension	1.79	1.32	2.43	1.79(1.32–2.43)	< 0.001	
			TyG_AIP ≤ median and hypertension	1.81	1.22	2.67	1.81(1.22–2.67)	0.003	
			TyG_AIP > median and hypertension	2.69	1.95	3.71	2.69(1.95–3.71)	< 0.001	
Yes	1895	32.8	TyG_AIP ≤ median and no hypertension	Reference					
			TyG_AIP > median and no hypertension	1.94	1.26	3	1.94(1.26–3.00)	0.003	
			TyG_AIP ≤ median and hypertension	2.22	1.34	3.68	2.22(1.34–3.68)	0.002	
			TyG_AIP > median and hypertension	3.71	2.38	5.8	3.71(2.38–5.80)	< 0.001	
Hukou									0.545
Rural	4736	81.9	TyG_AIP ≤ median and no hypertension	Reference					
			TyG_AIP > median and no hypertension	1.95	1.48	2.57	1.95(1.48–2.57)	< 0.001	
			TyG_AIP ≤ median and hypertension	2.05	1.47	2.87	2.05(1.47–2.87)	< 0.001	
			TyG_AIP > median and hypertension	2.93	2.18	3.93	2.93(2.18–3.93)	< 0.001	
Urban	1050	18.1	TyG_AIP ≤ median and no hypertension	Reference					
			TyG_AIP > median and no hypertension	1.41	0.79	2.55	1.41(0.79–2.55)	0.248	
			TyG_AIP ≤ median and hypertension	1.48	0.66	3.29	1.48(0.66–3.29)	0.339	
			TyG_AIP > median and hypertension	3.03	1.7	5.4	3.03(1.70–5.40)	< 0.001	
Marital_status									0.583
Married or partnered	5077	87.7	TyG_AIP ≤ median and no hypertension	Reference					
			TyG_AIP > median and no hypertension	1.97	1.5	2.58	1.97(1.50–2.58)	< 0.001	
			TyG_AIP ≤ median and hypertension	1.97	1.39	2.79	1.97(1.39–2.79)	< 0.001	
			TyG_AIP > median and hypertension	3.14	2.36	4.18	3.14(2.36–4.18)	< 0.001	
Separated	709	12.3	TyG_AIP ≤ median and no hypertension	Reference					
			TyG_AIP > median and no hypertension	1.22	0.63	2.36	1.22(0.63–2.36)	0.56	
			TyG_AIP ≤ median and hypertension	1.5	0.76	2.93	1.50(0.76–2.93)	0.24	
			TyG_AIP > median and hypertension	2.08	1.1	3.91	2.08(1.10–3.91)	0.024	
BMI									0.73
Abnormal BMI	2294	39.6	TyG_AIP ≤ median and no hypertension	Reference					
			TyG_AIP > median and no hypertension	1.71	1.13	2.58	1.71(1.13–2.58)	0.011	
			TyG_AIP ≤ median and hypertension	1.77	1.06	2.97	1.77(1.06–2.97)	0.03	
			TyG_AIP > median and hypertension	3.02	2	4.55	3.02(2.00–4.55)	< 0.001	
Normal BMI	3492	60.4	TyG_AIP ≤ median and no hypertension	Reference					
			TyG_AIP > median and no hypertension	1.86	1.36	2.57	1.86(1.36–2.57)	< 0.001	
			TyG_AIP ≤ median and hypertension	2.02	1.38	2.97	2.02(1.38–2.97)	< 0.001	
			TyG_AIP > median and hypertension	2.6	1.8	3.75	2.60(1.80–3.75)	< 0.001	
Age									0.133
> 60	2349	40.6	TyG_AIP ≤ median and no hypertension	Reference					
			TyG_AIP > median and no hypertension	1.87	1.3	2.7	1.87(1.30–2.70)	0.001	
			TyG_AIP ≤ median and hypertension	1.55	1.01	2.37	1.55(1.01–2.37)	0.043	
			TyG_AIP > median and hypertension	2.26	1.55	3.29	2.26(1.55–3.29)	< 0.001	
45–60	3437	59.4	TyG_AIP ≤ median and no hypertension	Reference					
			TyG_AIP > median and no hypertension	1.85	1.32	2.6	1.85(1.32–2.60)	< 0.001	
			TyG_AIP ≤ median and hypertension	2.25	1.43	3.53	2.25(1.43–3.53)	< 0.001	
			TyG_AIP > median and hypertension	3.63	2.53	5.22	3.63(2.53–5.22)	< 0.001	
Heart_problem									0.902
No	5028	86.9	TyG_AIP ≤ median and no hypertension	Reference					
			TyG_AIP > median and no hypertension	1.85	1.41	2.44	1.85(1.41–2.44)	< 0.001	
			TyG_AIP ≤ median and hypertension	2.01	1.43	2.82	2.01(1.43–2.82)	< 0.001	
			TyG_AIP > median and hypertension	2.91	2.17	3.9	2.91(2.17–3.90)	< 0.001	
Yes	758	13.1	TyG_AIP ≤ median and no hypertension	Reference					
			TyG_AIP > median and no hypertension	1.5	0.84	2.69	1.50(0.84–2.69)	0.171	
			TyG_AIP ≤ median and hypertension	1.52	0.72	3.18	1.52(0.72–3.18)	0.269	
			TyG_AIP > median and hypertension	2.55	1.42	4.57	2.55(1.42–4.57)	0.002	
Dyslipidemia									0.859
No	5164	89.2	TyG_AIP ≤ median and no hypertension	Reference					
			TyG_AIP > median and no hypertension	1.74	1.33	2.28	1.74(1.33–2.28)	< 0.001	
			TyG_AIP ≤ median and hypertension	1.82	1.3	2.55	1.82(1.30–2.55)	< 0.001	
			TyG_AIP > median and hypertension	2.54	1.89	3.42	2.54(1.89–3.42)	< 0.001	
Yes	622	10.8	TyG_AIP ≤ median and no hypertension	Reference					
			TyG_AIP > median and no hypertension	1.72	0.87	3.38	1.72(0.87–3.38)	0.117	
			TyG_AIP ≤ median and hypertension	2.35	1.05	5.24	2.35(1.05–5.24)	0.037	
			TyG_AIP > median and hypertension	2.95	1.52	5.71	2.95(1.52–5.71)	0.001	
Diabetes									0.883
No	5399	93.3	TyG_AIP ≤ median and no hypertension	Reference			4.7		
			TyG_AIP > median and no hypertension	1.8	1.39	2.32	8.2	< 0.001	
			TyG_AIP ≤ median and hypertension	1.92	1.4	2.64	8.8	< 0.001	
			TyG_AIP > median and hypertension	2.8	2.12	3.68	12.5	< 0.001	
Yes	387	6.7	TyG_AIP ≤ median and no hypertension	Reference			4.8		
			TyG_AIP > median and no hypertension	2.37	0.79	7.08	11	0.123	
			TyG_AIP ≤ median and hypertension	2.52	0.63	10.09	11.4	0.191	
			TyG_AIP > median and hypertension	4.26	1.47	12.3	18.7	0.008	

Boldface indicates statistically significant associations (p<0.05)

HR, hazard ratio; CI, confidence interval; TyG-AIP, combined triglyceride-glucose and atherogenic index of plasma; BMI, body mass index

Hazard ratios for stroke across population subgroups are presented. Participants were cross-classified into four exposure groups based on their TyG-AIP level (median as cutoff) and baseline hypertension status. Within each stratum (e.g., Sex), the group with “TyG-AIP ≤ median and no hypertension” was set as the reference. Associations were assessed using Cox proportional hazards models adjusted for age, sex, smoking, drinking, BMI, marital status, education, household registration (Hukou), cancer, heart disease, dyslipidemia, diabetes, anti-diabetic medication, cardiac medication, LDL-C, total cholesterol, and HbA1c (equivalent to the fully adjusted Model 3 described in the Methods). The P for interaction value tests whether the association between the four-level exposure variable and stroke risk differs significantly across the levels of the subgrouping variable (e.g., between males and females). The qualitatively similar shapes, reflecting shared biological pathways. The key distinction lies in the integrated index’s enhanced predictive utility (see below)

Age also modified the association in both the overall and PDM populations, with stronger effects observed in participants aged 45–60 years (*P*_interaction_ overall = 0.017; PDM = 0.013). These results collectively suggest that while the impact of TyG-AIP and hypertension on stroke risk is robust across populations, it may be more pronounced in certain subgroups, particularly middle-aged individuals.

## Discussion

This prospective cohort study examined the combined impact of the TyG-AIP index, its short-term dynamic changes, and hypertension on the risk of stroke. The study identified a significant non-linear relationship between TyG-AIP and stroke risk, particularly among individuals with abnormal glucose metabolism, where the risk rose sharply once the TyG-AIP threshold exceeded 3.386. More importantly, a pronounced joint association was observed between TyG-AIP and hypertension, indicating that the coexistence of these two metabolic disturbances substantially amplified stroke risk compared with either factor alone. This interaction remained consistent across different glucose metabolism states. Furthermore, trajectory analysis demonstrated that participants with a “high-level rapid-decline” TyG-AIP pattern were at significantly greater risk of stroke, underscoring the importance of temporal changes in metabolic-atherogenic status. The predictive modeling results further supported these findings, showing that a combined model incorporating both TyG-AIP trajectories and hypertension achieved the highest discriminatory accuracy for stroke prediction. Subgroup analysis also revealed that education level may modify these associations, suggesting that socioeconomic factors could interact with metabolic risk pathways. Collectively, these findings highlight the need to integrate both lipid-glucose metabolic dysregulation and blood pressure control into comprehensive stroke risk assessment strategies.

The TyG index, a composite biomarker derived from fasting triglycerides and glucose, has been widely recognized as a surrogate marker of insulin resistance and an early indicator of metabolic disorders, atherosclerosis, and cardiovascular events [29–31]. Previous studies have consistently shown that higher TyG index values are associated with increased risk of stroke incidence and mortality in both general and high-risk populations [6, 32–35]. However, despite its utility, the clinical application of the TyG index remains debated. On one hand, it can be affected by extreme triglyceride or glucose levels, potentially limiting its reliability in patients with severe hypertriglyceridemia or hyperglycemia [35]. On the other hand, traditional single indicators, such as fasting glucose or triglyceride levels, may provide more straightforward clinical interpretation [36]. Therefore, the TyG index alone might not fully capture the longitudinal complexity of metabolic dysregulation related to cerebrovascular risk.

Nevertheless, increasing evidence from large-scale clinical studies supports the TyG index as a valuable marker of insulin resistance and cardiometabolic risk. For example, research in young and middle-aged adults has demonstrated that the TyG index outperforms single metabolic indicators in predicting insulin resistance [37]. Findings from the VMCUN cohort further showed that the TyG index was superior to fasting glucose or triglycerides alone in predicting the development of type 2 diabetes among normoglycemic individuals [38]. Similarly, results from the Vascular Metabolism CUN cohort indicated that higher TyG index levels were independently associated with an elevated risk of cerebrovascular, peripheral arterial, and coronary heart diseases [39]. Together, this evidence strengthens the rationale for incorporating TyG-related indices, such as TyG-AIP, into stroke risk prediction frameworks.

The AIP overcomes the limitation that single lipid parameters cannot fully capture the risk of cardiovascular and cerebrovascular diseases [40, 41]. Calculated from the ratio of triglycerides to HDL-C, AIP has been widely recognized as an important marker for predicting various adverse cardiometabolic and cerebrovascular outcomes [40, 41]. Numerous studies have demonstrated that AIP is significantly associated with metabolic syndrome, hypertension, and diabetes, and is closely linked to the onset and poor prognosis of cardiovascular and cerebrovascular events [42, 43]. For instance, one study examining the relationship between AIP and incident stroke across different glucose metabolism statuses found that higher baseline AIP levels were associated with an increased risk of stroke among middle-aged and elderly individuals, with variations observed across glycemic categories [44].This is consistent with our findings that elevated AIP levels are associated with a higher stroke risk under dysglycemic conditions, further supporting the validity of our results. Moreover, evidence from the Kailuan Study indicated that a higher cumulative AIP was associated with an increased risk of ischemic stroke [43], while several prospective cohorts have confirmed positive correlations between AIP and stroke risk in individuals with cardiorenal metabolic syndrome stages 0–3 [45, 46].

Given the shared metabolic pathways between the TyG index and AIP, we combined these two indices to derive the TyG-AIP index, aiming to capture both glucose-lipid metabolic disturbances and atherogenic risk more comprehensively. Multivariable Cox regression analysis revealed a significant association between the TyG-AIP index and stroke risk, particularly among individuals with abnormal glucose metabolism. This finding aligns with previous evidence suggesting that both TyG and AIP independently contribute to stroke risk in populations with dysglycemia, highlighting the potential of TyG-AIP as an integrated marker of metabolic-atherogenic burden.

Although the biological mechanisms linking TyG-AIP to stroke risk are not yet fully elucidated, several plausible pathways have been proposed. Insulin resistance, a core metabolic abnormality reflected by TyG-related indices, can promote endothelial dysfunction at the early stages of atherosclerosis, activate inflammatory cascades, enhance foam cell formation, stimulate vascular smooth muscle proliferation, and accelerate plaque progression through apoptosis of vascular smooth muscle cells [47, 48]. The TyG index also captures disturbances in glucose metabolism, oxidative stress, and subclinical inflammation [49]. Furthermore, elevated TyG levels may reflect dysregulation in advanced glycation end-product metabolism and enhanced platelet reactivity, both of which impair endothelium-dependent vasodilation [50]. Additionally, higher TyG values are often accompanied by elevated circulating free fatty acids, which further exacerbate insulin resistance and vascular injury [49]. Collectively, these mechanisms provide a biological basis for the observed relationship between elevated TyG-AIP levels and increased stroke risk.

The AIP is closely associated with lipoprotein particle size, particularly showing a negative correlation with LDL particle size. Since smaller, denser LDL particles are a well-established risk factor for cardiovascular and cerebrovascular events [51, 52], AIP demonstrates strong predictive value for stroke. Furthermore, AIP reflects long-term atherosclerotic risk and lipid metabolism disorders. Dysregulated lipid metabolism can lead to visceral fat accumulation, ultimately contributing to central obesity [53–55]. Visceral adipose tissue, in turn, impairs insulin signaling by releasing pro-inflammatory cytokines such as IL-6, promoting insulin resistance and glucose metabolism disturbances.

Additionally, elevated AIP is closely associated with endothelial dysfunction, where high triglyceride levels and low HDL-C levels compromise endothelial cell function, decrease nitric oxide production, and impair vasodilation, leading to increased vascular resistance and a heightened risk of hypertension and stroke [56, 57]. The TyG-AIP index, which combines these markers, further strengthens its predictive ability for stroke risk, particularly in individuals with impaired glucose metabolism.

Moreover, our findings indicated that stroke risk remained significant in the “high-level rapid-decline” TyG-AIP trajectory group, suggesting that the extent of vascular damage is strongly influenced by the level of metabolic exposure. Short-term improvements in TyG-AIP levels are insufficient to immediately reverse vascular injury caused by prolonged high exposure [58]. Persistent high TyG-AIP levels have likely led to the formation of unstable atherosclerotic plaques [59]. Chronic metabolic stress, including long-term insulin resistance, hyperglycemia, and lipotoxicity, induces epigenetic modifications and persistent functional damage to endothelial and smooth muscle cells, further contributing to vascular instability [59]. Furthermore, prior to stroke onset, patients’ pre-existing subclinical conditions may lead to malnutrition and impaired nutrient absorption, which can alter blood glucose and triglyceride levels.

Our analysis further demonstrates that a model combining TyG-AIP with hypertension offers superior discrimination of stroke risk compared to models using either factor alone (Fig. 7). This improvement in predictive accuracy serves as a key justification for considering these variables jointly. It underscores the clinical utility of integrating metabolic and hemodynamic profiles for more effective risk stratification, aligning with their complementary roles in stroke pathophysiology. The observed improvement in predictive accuracy is supported by a strong pathophysiological rationale. Insulin resistance (reflected by TyG-AIP) and hypertension are known to interact through mechanisms such as sympathetic activation and endothelial dysfunction, creating a vicious cycle that accelerates cardiovascular risk. Therefore, even in the absence of a statistically multiplicative interaction, their co-occurrence defines a distinct, high-risk clinical phenotype. From a public health perspective, our findings quantitatively identify this “dual-burden” subgroup as warranting prioritized, integrated management strategies targeting both metabolic health and blood pressure control. We have enhanced the Discussion section to elaborate on these points.

In summary, a high-level declining TyG-AIP trajectory is an independent risk factor for stroke. Insulin resistance, endothelial dysfunction, and reduced nitric oxide bioavailability under this prolonged high-level state promote vasoconstriction and the development of hypertension. Additionally, LDL particles exposed to prolonged high metabolic stress are more prone to oxidation, leading to macrophage uptake and foam cell formation, thereby promoting atherosclerotic plaque development [60]. Chronic inflammation further destabilizes the vascular endothelium, fostering thrombus formation and increasing stroke risk. rtension is the most common modifiable risk factor for stroke and plays a critical role in its onset and progression [12, 61]. In our preliminary analysis of the TyG-AIP index and stroke risk, we observed that the most significant difference in stroke risk was based on the presence or absence of hypertension. Consequently, we incorporated hypertension into our analysis to assess its impact on stratified stroke prevention and risk prediction. Our findings confirmed that the combination of the TyG-AIP index (and its trajectories) with hypertension resulted in a significantly higher stroke risk, while also enhancing the predictive accuracy for stroke [37]. This joint association is closely related to the cumulative risk factors associated with both hypertension and TyG-AIP, such as chronic inflammation, insulin resistance, and central obesity [62].

Our stratified analyses revealed that hypertension modifies the association between TyG-AIP trajectories and cardiovascular risk in complex ways that differ by glycemic status. In normoglycemic individuals, the presence of hypertension appears to dominate the risk profile, with unfavorable TyG-AIP trajectories conferring little additional risk. This may reflect a “ceiling effect” where the pathophysiological impact of hypertension on vascular endothelial function, arterial stiffness, and hemodynamic stress overwhelms the more subtle metabolic disturbances captured by TyG-AIP trajectories.

Conversely, in prediabetes, both hypertension and unfavorable TyG-AIP trajectories contribute additively to risk. This differential pattern may stem from the more advanced underlying metabolic dysfunction in prediabetes, where insulin resistance, dyslipidemia, and hyperglycemia create a synergistic milieu with hypertension through shared mechanisms including oxidative stress, inflammation, and endothelial dysfunction.

These findings have important clinical implications. For normoglycemic hypertensive patients, aggressive blood pressure control should remain the primary focus, as it addresses the dominant risk factor. For prediabetic individuals, however, comprehensive management addressing both metabolic parameters and blood pressure is warranted, as both contribute independently to cardiovascular risk.

Our ROC analyses revealed that TyG-AIP does not provide statistically superior discrimination for stroke compared to its individual components or hypertension alone. These modest AUC values (0.571–0.590) reflect the inherent limitations of single biomarkers in predicting multifactorial diseases like stroke, where individual risk factors typically exhibit limited discriminative capacity when considered in isolation. However, this finding should not obscure the primary contribution of our study, which lies in elucidating the joint effects and interactions between TyG-AIP trajectories and hypertension. Rather than serving as a standalone predictive tool, TyG-AIP appears most valuable as an integrative metabolic indicator that, when considered alongside hypertension status, helps identify high-risk subgroups (HR up to 3.38). This suggests that clinical risk assessment may benefit more from evaluating the confluence of metabolic and hemodynamic risk factors than from comparing the predictive performance of individual biomarkers.

Our examination of the interplay between TyG-AIP and hypertension yielded nuanced insights. A multiplicative interaction term was statistically significant in the total cohort but not within glycemic subgroups, and its direction suggested attenuation of the TyG-AIP effect in hypertensives. This may reflect methodological scale-dependence or a ‘ceiling effect’ of a potent risk factor. Irrespective of this statistical nuance, the clinical implications are unequivocal. The joint exposure analysis consistently identified individuals with both high TyG-AIP and hypertension as the highest-risk group across all populations. Furthermore, the TyG-AIP index provided significant incremental value for risk reclassification (NRI). Therefore, the principal clinical utility of TyG-AIP lies in its ability to integrate metabolic and hypertensive risk into a single metric that effectively identifies patients at the greatest absolute risk of stroke, a finding robust across different metabolic states.

### Addressing the similarity of dose–response curves

The restricted cubic spline analyses revealed qualitatively similar non-linear relationships between TyG, AIP, TyG-AIP, and stroke risk (Fig. 5). This observation is biologically plausible and mathematically expected, as both TyG and AIP are derived from overlapping lipid and glucose metabolism pathways, and TyG-AIP is a composite index integrating these two components. Therefore, the primary contribution of TyG-AIP is not to reveal a novel risk curve shape, but to synthesize two interrelated yet distinct pathophysiological processes—insulin resistance (captured by TyG) and atherogenic dyslipidemia (captured by AIP)—into a single, clinically pragmatic metric. The value of this integration is demonstrated empirically in our study: First, the integrated TyG-AIP index showed a stronger joint effect with hypertension than either TyG or AIP alone, identifying a distinct high-risk phenotype. Second, and most importantly, adding TyG-AIP to a conventional risk model (age, sex, hypertension) resulted in a significant improvement in net risk reclassification (continuous NRI = 0.193), quantifiably proving its incremental utility at the individual level. Thus, while the curves are similar, TyG-AIP provides superior integrative capacity, statistical robustness, and clinical applicability for comprehensive metabolic risk assessment.

Our study demonstrates that the integrated TyG-AIP and hypertension model not only improves the discriminative ability for stroke (as shown by AUC) but also significantly enhances the accuracy of individual risk stratification, as evidenced by the positive Net Reclassification Index across all analyzed populations. The significant NRI, particularly driven by the correct downward reclassification of individuals who did not experience a stroke, suggests that this model could help refine primary prevention strategies by more accurately identifying those at truly high risk, while reducing unnecessary interventions in lower-risk individuals. This underscores the translational potential of combining metabolic indices with hypertension status in routine clinical risk assessment.

The strengths of this study are as follows. First, this is the first nationally representative prospective cohort study to investigate the association between the TyG-AIP index, its trajectories, and stroke risk, and to reveal its synergistic predictive effect in combination with hypertension. The strong representativeness of the cohort and the long follow-up period contribute to the robustness and reliability of the findings. Second, this study employed multivariable Cox regression analysis, supplemented by subgroup and sensitivity analyses, to thoroughly evaluate both the independent and joint effects of the TyG-AIP index and hypertension on stroke risk. The use of multiple statistical approaches strengthens the validity and reliability of the results. Additionally, by utilizing restricted cubic splines, we identified a non-linear relationship between TyG-AIP and stroke risk across different glucose metabolism statuses. Threshold analysis further revealed that individuals with dysglycemia and a TyG-AIP > 3.386 should be considered at high risk for stroke, regardless of their specific glycemic status, and should be managed accordingly. This study employed two complementary approaches. On the one hand, traditional quantile analysis reaffirmed TyG-AIP as a potent static risk marker. On the other hand, and more innovatively, trajectory clustering revealed that the dynamic change in TyG-AIP carries independent prognostic value. For instance, the “high-level rapid decline” trajectory was associated with a 36% higher stroke risk compared to the “low-level gradual decline” group (HR = 1.36, 95% CI 1.03–1.79), suggesting that a history of high exposure confers residual risk even during a declining phase. The observed overlap between clusters in Fig. 2 likely reflects the continuous nature of metabolic dysregulation in real-world populations rather than invalid groupings, as evidenced by their distinct and clinically significant risk profiles.Moreover, given the easy accessibility and measurability of the TyG-AIP index and hypertension, the findings from this study can be readily applied to public health screening and chronic disease intervention. These results provide valuable scientific evidence for disease prediction and have significant implications for implementing preventive strategies in high-risk populations.

Our study has limitations. The clustering solution, while clinically informative and validated by outcome differences, showed moderate internal cohesion (silhouette coefficient = 0.479) and visual overlap, which is characteristic of phenotypes existing on a biological spectrum. Future studies with more frequent measurements could refine these trajectory definitions. A potential limitation is the relatively small number of cardiovascular events within the prediabetes and diabetes subgroups, which limited our ability to perform robust stratified analyses as suggested in the literature. Instead, we formally tested for interaction effects, finding no evidence that glycemic status modifies the association between TyG-AIP and outcomes (see Supplementary Table 15). While this approach addresses the question of effect modification, larger studies specifically powered for subgroup analyses within dysglycemia populations would provide more definitive evidence. This study focused on a core set of traditional and metabolic risk factors. While this provides clear insights into the roles of TyG-AIP and hypertension, it did not incorporate other potential biomarkers (e.g., inflammatory markers like C-reactive protein, or detailed renal function parameters). Future research integrating a broader panel of biomarkers could further refine the risk stratification model. Fourth, our ROC analyses demonstrated that TyG-AIP’s discriminative ability (AUC 0.590) was modest and not statistically superior to its components or hypertension. While this may limit its utility as a standalone screening tool, it does not negate the value of TyG-AIP as an integrative metabolic indicator or the importance of our findings regarding its interaction with hypertension. Future studies should explore whether incorporating TyG-AIP into comprehensive risk prediction models or evaluating its role in risk reclassification might yield greater clinical utility.

## Conclusion

Our study demonstrates that unfavorable TyG-AIP trajectories are associated with increased stroke risk and exhibit significant joint effects with hypertension. Although ROC analyses indicated that TyG-AIP alone does not provide statistically superior discrimination compared to its individual components, its integration into a comprehensive risk assessment framework—particularly when considered alongside hypertension—identifies individuals at substantially elevated risk. This joint effect remains consistent across populations with different glucose metabolism statuses, while education level serves as an important modifier of this association. Therefore, in primary stroke prevention, a holistic approach that combines blood pressure control with monitoring of TyG-AIP trajectories may enhance risk stratification. Specifically, middle-aged individuals presenting with both elevated TyG-AIP trajectories and hypertension warrant more intensive intervention strategies.

## Supplementary Information

Below is the link to the electronic supplementary material.

Supplementary Material 1

## Data Availability

The original contributions presented in the study are included in the manuscript. Further inquiries can be directed to the corresponding author.

## Abbreviations

TyG: Triglyceride-glucose index
AIP: Atherogenic index of plasma
PDM: Dysglycemia
NDM: Normoglycemia
ROC: Receiver operating characteristic
DALYs: Disability-adjusted life years
IR: Insulin resistance
TG: Triglycerides
HDL-C: High-density lipoprotein cholesterol
BMI: Body mass index
HbA1c: Glycated hemoglobin
FBG: Fasting blood glucose
LDL-C: Low-density lipoprotein cholesterol
HRs: Hazard ratios
CIs: Confidence intervals
MICE: Multiple imputation by chained equations
IQR: Interquartile range
SD: Standard deviation
RCS: Restricted cubic spline
NRI: Net reclassification index
IDI: Integrated discrimination improvement
