# Supplementary material for: The joint association of the combined triglyceride-glucose index and atherogenic index of plasma with hypertension on stroke risk across different glycemic status: a prospective cohort study
Source: Cardiovasc Diabetol. 2026 Mar 26;25:141. doi: 10.1186/s12933-026-03080-9 (PMC13147867; doi:10.1186/s12933-026-03080-9)
Supplement: Supplementary file 1 — Supplementary Material 1 [file 12933_2026_3080_MOESM1_ESM.docx]

**Supplemental Table**

**Supplemental table1: Subgroup analysis in the total population stratified by the presence or absence of hypertension.**

| **Variable** | **Count** | **Percent** | **Point Estimate** | **Lower** | **Upper** | **No hypertension** | **Hypertension** | **P value** | **P for interaction** |
| --- | --- | --- | --- | --- | --- | --- | --- | --- | --- |
| Overall | 5786 | 100 | 1.81 | 1.5 | 2.18 | 6.5 | 11.4 | <0.001 |  |
| Sex |  |  |  |  |  |  |  |  | 0.163 |
| Female | 3124 | 54 | 1.6 | 1.23 | 2.07 | 6.5 | 10.2 | <0.001 |  |
| Male | 2662 | 46 | 2.07 | 1.59 | 2.7 | 6.4 | 12.8 | <0.001 |  |
| Education |  |  |  |  |  |  |  |  | 0.48 |
| Below high school | 5153 | 89.1 | 1.77 | 1.46 | 2.15 | 6.6 | 11.4 | <0.001 |  |
| High school or above | 633 | 10.9 | 2.21 | 1.22 | 3.99 | 5.5 | 11.8 | 0.009 |  |
| Smoking |  |  |  |  |  |  |  |  | 0.517 |
| No | 3507 | 60.6 | 1.72 | 1.35 | 2.2 | 6.2 | 10.5 | <0.001 |  |
| Yes | 2279 | 39.4 | 1.94 | 1.46 | 2.56 | 6.8 | 12.8 | <0.001 |  |
| Drinking |  |  |  |  |  |  |  |  | 0.251 |
| No | 3891 | 67.2 | 1.68 | 1.33 | 2.11 | 6.5 | 10.7 | <0.001 |  |
| Yes | 1895 | 32.8 | 2.1 | 1.53 | 2.88 | 6.4 | 12.9 | <0.001 |  |
| Hukou |  |  |  |  |  |  |  |  | 0.558 |
| Rural | 4736 | 81.9 | 1.76 | 1.43 | 2.16 | 6.5 | 11.1 | <0.001 |  |
| Urban | 1050 | 18.1 | 2.02 | 1.33 | 3.07 | 6.5 | 12.7 | 0.001 |  |
| Marital status |  |  |  |  |  |  |  |  | 0.657 |
| Married or partnered | 5077 | 87.7 | 1.81 | 1.48 | 2.22 | 6.2 | 11 | <0.001 |  |
| Separated | 709 | 12.3 | 1.61 | 1.02 | 2.55 | 8.5 | 13.2 | 0.04 |  |
| Dyslipidemia |  |  |  |  |  |  |  |  | 0.527 |
| No | 5164 | 89.2 | 1.65 | 1.34 | 2.04 | 5.9 | 9.5 | <0.001 |  |
| Yes | 622 | 10.8 | 1.9 | 1.29 | 2.8 | 12.5 | 22.4 | 0.001 |  |
| Diabetes |  |  |  |  |  |  |  |  | 0.579 |
| No | 5399 | 93.3 | 1.75 | 1.44 | 2.13 | 6.3 | 10.8 | <0.001 |  |
| Yes | 387 | 6.7 | 2.08 | 1.17 | 3.7 | 8.7 | 17.1 | 0.013 |  |
| BMI |  |  |  |  |  |  |  |  | 0.722 |
| Normal BMI | 2294 | 39.6 | 1.82 | 1.4 | 2.38 | 7.4 | 13.1 | <0.001 |  |
| Abnormal BMI | 3492 | 60.4 | 1.7 | 1.31 | 2.21 | 5.9 | 9.9 | <0.001 |  |
| Age |  |  |  |  |  |  |  |  | 0.017 |
| 45-60 | 3437 | 59.4 | 2.17 | 1.67 | 2.82 | 5.4 | 11.4 | <0.001 |  |
| >60 | 2349 | 40.6 | 1.38 | 1.06 | 1.8 | 8.4 | 11.4 | 0.015 |  |
| Heart_problem |  |  |  |  |  |  |  |  | 0.769 |
| No | 5028 | 86.9 | 1.8 | 1.46 | 2.22 | 5.9 | 10.4 | <0.001 |  |
| Yes | 758 | 13.1 | 1.68 | 1.12 | 2.5 | 10.4 | 16.8 | 0.012 |  |

**Supplemental table 2:Subgroup analysis in the population with abnormal glucose metabolism stratified by the presence or absence of hypertension.**

| **Variable** | **Count** | **Percent** | **Point Estimate** | **Lower** | **Upper** | **No hypertension** | **Hypertension** | **P value** | **P for interaction** |
| --- | --- | --- | --- | --- | --- | --- | --- | --- | --- |
| Overall | 3490 | 100 | 1.67 | 1.34 | 2.09 | 7.3 | 11.9 | <0.001 |  |
| Sex |  |  |  |  |  |  |  |  | 0.502 |
| Female | 1875 | 53.7 | 1.55 | 1.13 | 2.14 | 6.9 | 10.5 | 0.007 |  |
| Male | 1615 | 46.3 | 1.8 | 1.32 | 2.47 | 7.7 | 13.6 | <0.001 |  |
| Education |  |  |  |  |  |  |  |  | 0.681 |
| Below high school | 3126 | 89.6 | 1.7 | 1.34 | 2.16 | 7.2 | 11.9 | <0.001 |  |
| High school or above | 364 | 10.4 | 1.46 | 0.73 | 2.91 | 8.3 | 11.8 | 0.287 |  |
| Smoking |  |  |  |  |  |  |  |  | 0.743 |
| No | 2120 | 60.7 | 1.73 | 1.29 | 2.32 | 6.9 | 11.6 | <0.001 |  |
| Yes | 1370 | 39.3 | 1.6 | 1.13 | 2.26 | 8 | 12.4 | 0.008 |  |
| Drinking |  |  |  |  |  |  |  |  | 0.499 |
| No | 2344 | 67.2 | 1.58 | 1.19 | 2.09 | 7.1 | 11 | 0.001 |  |
| Yes | 1146 | 32.8 | 1.85 | 1.28 | 2.69 | 7.7 | 13.8 | 0.001 |  |
| Hukou |  |  |  |  |  |  |  |  | 0.691 |
| Rural | 2795 | 80.1 | 1.63 | 1.27 | 2.1 | 7.3 | 11.6 | <0.001 |  |
| Urban | 695 | 19.9 | 1.82 | 1.12 | 2.96 | 7.4 | 13.1 | 0.016 |  |
| Marital status |  |  |  |  |  |  |  |  | 0.553 |
| Married or partnered | 3038 | 87 | 1.62 | 1.27 | 2.07 | 7.4 | 11.7 | <0.001 |  |
| Separated | 452 | 13 | 1.97 | 1.08 | 3.62 | 6.9 | 13.1 | 0.028 |  |
| Dyslipidemia |  |  |  |  |  |  |  |  | 0.219 |
| No | 3038 | 87 | 1.46 | 1.13 | 1.9 | 6.7 | 9.7 | 0.004 |  |
| Yes | 452 | 13 | 2.03 | 1.28 | 3.2 | 12.1 | 23 | 0.003 |  |
| Diabetes |  |  |  |  |  |  |  |  | 0.374 |
| No | 3140 | 90 | 1.59 | 1.25 | 2.02 | 7.2 | 11.2 | <0.001 |  |
| Yes | 350 | 10 | 2.15 | 1.15 | 4 | 8.3 | 16.7 | 0.016 |  |
| BMI |  |  |  |  |  |  |  |  | 0.254 |
| Abnormal BMI | 1510 | 43.3 | 1.86 | 1.35 | 2.55 | 7.7 | 13.8 | <0.001 |  |
| Normal BMI | 1980 | 56.7 | 1.42 | 1.03 | 1.97 | 7.1 | 9.9 | 0.034 |  |
| Age |  |  |  |  |  |  |  |  | 0.013 |
| >60 | 1473 | 42.2 | 1.21 | 0.88 | 1.67 | 9.6 | 11.6 | 0.231 |  |
| 45-60 | 2017 | 57.8 | 2.14 | 1.55 | 2.93 | 6 | 12.3 | <0.001 |  |
| Heart_problem |  |  |  |  |  |  |  |  | 0.489 |
| No | 2999 | 85.9 | 1.71 | 1.33 | 2.2 | 6.7 | 11.2 | <0.001 |  |
| Yes | 491 | 14.1 | 1.4 | 0.86 | 2.29 | 11.4 | 15.5 | 0.179 |  |

**Supplemetal table 3: Subgroup analysis in the population with normal glucose metabolism stratified by the presence or absence of hypertension.**

| **Variable** | **Count** | **Percent** | **Point Estimate** | **Lower** | **Upper** | **No hypertension** | **Hypertension** | **P value** | **P for interaction** |
| --- | --- | --- | --- | --- | --- | --- | --- | --- | --- |
| Overall | 2296 | 100 | 1.99 | 1.44 | 2.76 | 5.3 | 10.3 | <0.001 |  |
| Sex |  |  |  |  |  |  |  |  | 0.161 |
| Female | 1249 | 54.4 | 1.62 | 1.03 | 2.53 | 6 | 9.6 | 0.035 |  |
| Male | 1047 | 45.6 | 2.59 | 1.59 | 4.22 | 4.5 | 11.2 | <0.001 |  |
| Education |  |  |  |  |  |  |  |  | 0.087 |
| Below high school | 2027 | 88.3 | 1.81 | 1.29 | 2.55 | 5.8 | 10.2 | 0.001 |  |
| High school or above | 269 | 11.7 | 5.37 | 1.64 | 17.59 | 2.3 | 11.8 | 0.006 |  |
| Smoking |  |  |  |  |  |  |  |  | 0.092 |
| No | 1387 | 60.4 | 1.52 | 0.96 | 2.41 | 5.3 | 8 | 0.072 |  |
| Yes | 909 | 39.6 | 2.68 | 1.66 | 4.31 | 5.3 | 13.6 | <0.001 |  |
| Drinking |  |  |  |  |  |  |  |  | 0.35 |
| No | 1547 | 67.4 | 1.79 | 1.21 | 2.66 | 5.7 | 10 | 0.004 |  |
| Yes | 749 | 32.6 | 2.51 | 1.4 | 4.49 | 4.6 | 11.1 | 0.002 |  |
| Hukou |  |  |  |  |  |  |  |  | 0.597 |
| Rural | 1941 | 84.5 | 1.92 | 1.34 | 2.74 | 5.4 | 10.1 | <0.001 |  |
| Urban | 355 | 15.5 | 2.44 | 1.07 | 5.55 | 4.9 | 11.5 | 0.034 |  |
| Marital status |  |  |  |  |  |  |  |  | 0.204 |
| Married or partnered | 2039 | 88.8 | 2.07 | 1.43 | 3 | 4.8 | 9.6 | <0.001 |  |
| Separated | 257 | 11.2 | 1.23 | 0.61 | 2.5 | 11.1 | 13.5 | 0.561 |  |
| Dyslipidemia |  |  |  |  |  |  |  |  | 0.611 |
| No | 2126 | 92.6 | 1.97 | 1.37 | 2.83 | 4.8 | 9.2 | <0.001 |  |
| Yes | 170 | 7.4 | 1.58 | 0.74 | 3.38 | 13.5 | 20.3 | 0.237 |  |
| Diabetes |  |  |  |  |  |  |  |  | 0.869 |
| No | 2259 | 98.4 | 1.97 | 1.41 | 2.76 | 5.2 | 10.1 | <0.001 |  |
| Yes | 37 | 1.6 | 1.69 | 0.34 | 8.38 | 13 | 21.4 | 0.52 |  |
| BMI |  |  |  |  |  |  |  |  | 0.349 |
| Abnormal BMI | 784 | 34.1 | 1.63 | 0.98 | 2.71 | 6.9 | 11 | 0.06 |  |
| Normal BMI | 1512 | 65.9 | 2.24 | 1.46 | 3.43 | 4.6 | 9.9 | <0.001 |  |
| Age |  |  |  |  |  |  |  |  | 0.578 |
| >60 | 1420 | 61.8 | 2.09 | 1.32 | 3.32 | 4.7 | 9.7 | 0.002 |  |
| 45-60 | 876 | 38.2 | 1.73 | 1.08 | 2.77 | 6.5 | 11 | 0.022 |  |
| Heart_problem |  |  |  |  |  |  |  |  | 0.523 |
| No | 2029 | 88.4 | 1.85 | 1.28 | 2.69 | 4.9 | 8.9 | 0.001 |  |
| Yes | 267 | 11.6 | 2.4 | 1.2 | 4.81 | 8.9 | 20 | 0.013 |  |

**Supplemental table 4: Subgroup analysis in the total population stratified by TyG-AIP levels.**

| **Variable** | **Count** | **Percent** | **Point Estimate** | **Lower** | **Upper** | **TyG-AIP ≤ median** | **TyG-AIP > median** | **P value** | **P for interaction** |
| --- | --- | --- | --- | --- | --- | --- | --- | --- | --- |
| Overall | 5786 | 100 | 1.78 | 1.47 | 2.15 | 5.8 | 10.1 | <0.001 |  |
| Sex |  |  |  |  |  |  |  |  | 0.189 |
| Female | 3124 | 54 | 1.59 | 1.22 | 2.06 | 5.9 | 9.2 | 0.001 |  |
| Male | 2662 | 46 | 2.04 | 1.55 | 2.68 | 5.7 | 11.2 | <0.001 |  |
| Education |  |  |  |  |  |  |  |  | 0.082 |
| Below high school | 5153 | 89.1 | 1.69 | 1.39 | 2.06 | 6.1 | 10.1 | <0.001 |  |
| High school or above | 633 | 10.9 | 3.31 | 1.6 | 6.88 | 3.2 | 10.2 | 0.001 |  |
| Smoking |  |  |  |  |  |  |  |  | 0.615 |
| No | 3507 | 60.6 | 1.72 | 1.34 | 2.21 | 5.6 | 9.4 | <0.001 |  |
| Yes | 2279 | 39.4 | 1.89 | 1.41 | 2.52 | 6.2 | 11.3 | <0.001 |  |
| Drinking |  |  |  |  |  |  |  |  | 0.666 |
| No | 3891 | 67.2 | 1.74 | 1.37 | 2.2 | 5.7 | 9.7 | <0.001 |  |
| Yes | 1895 | 32.8 | 1.89 | 1.37 | 2.61 | 6 | 11 | <0.001 |  |
| Hukou |  |  |  |  |  |  |  |  | 0.951 |
| Rural | 4736 | 81.9 | 1.79 | 1.45 | 2.2 | 5.8 | 10.1 | <0.001 |  |
| Urban | 1050 | 18.1 | 1.75 | 1.11 | 2.76 | 6 | 10.2 | 0.015 |  |
| Marital status |  |  |  |  |  |  |  |  | 0.15 |
| Married or partnered | 5077 | 87.7 | 1.91 | 1.55 | 2.35 | 5.3 | 9.9 | <0.001 |  |
| Separated | 709 | 12.3 | 1.32 | 0.83 | 2.08 | 9.2 | 11.8 | 0.24 |  |
| Dyslipidemia |  |  |  |  |  |  |  |  | 0.797 |
| No | 5164 | 89.2 | 1.65 | 1.34 | 2.04 | 5.3 | 8.6 | <0.001 |  |
| Yes | 622 | 10.8 | 1.54 | 0.98 | 2.44 | 12.4 | 18.5 | 0.063 |  |
| Diabetes |  |  |  |  |  |  |  |  | 0.501 |
| No | 5399 | 93.3 | 1.71 | 1.4 | 2.08 | 5.8 | 9.6 | <0.001 |  |
| Yes | 387 | 6.7 | 2.24 | 1.05 | 4.79 | 6.7 | 14.6 | 0.038 |  |
| BMI |  |  |  |  |  |  |  |  | 0.797 |
| Abnormal BMI | 2294 | 39.6 | 1.77 | 1.31 | 2.39 | 6.5 | 11.2 | <0.001 |  |
| Normal BMI | 3492 | 60.4 | 1.68 | 1.3 | 2.16 | 5.5 | 9 | <0.001 |  |
| Age |  |  |  |  |  |  |  |  | 0.632 |
| >60 | 2349 | 40.6 | 1.71 | 1.31 | 2.24 | 7.2 | 11.9 | <0.001 |  |
| 45-60 | 3437 | 59.4 | 1.88 | 1.44 | 2.45 | 4.8 | 8.9 | <0.001 |  |
| Heart_problem |  |  |  |  |  |  |  |  | 0.75 |
| No | 5028 | 86.9 | 1.75 | 1.42 | 2.17 | 5.4 | 9.2 | <0.001 |  |
| Yes | 758 | 13.1 | 1.62 | 1.05 | 2.5 | 9.4 | 14.9 | 0.031 |  |

**Supplemental table 5: Subgroup analysis in the population with abnormal glucose metabolism stratified by TyG-AIP levels.**

| **Variable** | **Count** | **Percent** | **Point Estimate** | **Lower** | **Upper** | **TyG-AIP ≤ median** | **TyG-AIP > median** | **P value** | **P for interaction** |
| --- | --- | --- | --- | --- | --- | --- | --- | --- | --- |
| Overall | 3490 | 100 | 1.62 | 1.29 | 2.03 | 6.9 | 10.8 | <0.001 |  |
| Sex |  |  |  |  |  |  |  |  | 0.11 |
| Female | 1875 | 53.7 | 1.36 | 0.98 | 1.88 | 6.9 | 9.2 | 0.064 |  |
| Male | 1615 | 46.3 | 1.96 | 1.42 | 2.72 | 6.8 | 12.9 | <0.001 |  |
| Education |  |  |  |  |  |  |  |  | 0.171 |
| Below high school | 3126 | 89.6 | 1.53 | 1.2 | 1.94 | 7.1 | 10.6 | 0.001 |  |
| High school or above | 364 | 10.4 | 2.71 | 1.23 | 5.99 | 5 | 12.8 | 0.014 |  |
| Smoking |  |  |  |  |  |  |  |  | 0.47 |
| No | 2120 | 60.7 | 1.51 | 1.12 | 2.04 | 6.8 | 10.1 | 0.007 |  |
| Yes | 1370 | 39.3 | 1.79 | 1.25 | 2.55 | 7 | 12.1 | 0.001 |  |
| Drinking |  |  |  |  |  |  |  |  | 0.96 |
| No | 2344 | 67.2 | 1.64 | 1.23 | 2.19 | 6.5 | 10.3 | 0.001 |  |
| Yes | 1146 | 32.8 | 1.62 | 1.11 | 2.35 | 7.7 | 12 | 0.012 |  |
| Hukou |  |  |  |  |  |  |  |  | 0.823 |
| Rural | 2795 | 80.1 | 1.59 | 1.23 | 2.06 | 6.9 | 10.7 | <0.001 |  |
| Urban | 695 | 19.9 | 1.7 | 1.01 | 2.85 | 6.9 | 11.3 | 0.047 |  |
| Marital status |  |  |  |  |  |  |  |  | 0.758 |
| Married or partnered | 3038 | 87 | 1.64 | 1.28 | 2.11 | 6.7 | 10.8 | <0.001 |  |
| Separated | 452 | 13 | 1.48 | 0.81 | 2.71 | 7.9 | 11.4 | 0.2 |  |
| Dyslipidemia |  |  |  |  |  |  |  |  | 0.922 |
| No | 3038 | 87 | 1.48 | 1.14 | 1.91 | 6.3 | 9.2 | 0.003 |  |
| Yes | 452 | 13 | 1.52 | 0.9 | 2.55 | 12.8 | 18.8 | 0.116 |  |
| Diabetes |  |  |  |  |  |  |  |  | 0.046 |
| No | 3140 | 90 | 1.46 | 1.15 | 1.86 | 7.1 | 10.1 | 0.002 |  |
| Yes | 350 | 10 | 3.91 | 1.54 | 9.98 | 4.2 | 15.6 | 0.004 |  |
| BMI |  |  |  |  |  |  |  |  | 0.869 |
| Abnormal BMI | 1510 | 43.3 | 1.6 | 1.13 | 2.26 | 7.6 | 11.8 | 0.008 |  |
| Normal BMI | 1980 | 56.7 | 1.53 | 1.12 | 2.1 | 6.5 | 9.8 | 0.008 |  |
| Age |  |  |  |  |  |  |  |  | 0.724 |
| >60 | 1473 | 42.2 | 1.58 | 1.15 | 2.17 | 8.4 | 12.8 | 0.005 |  |
| 45-60 | 2017 | 57.8 | 1.71 | 1.23 | 2.38 | 5.7 | 9.5 | 0.001 |  |
| Heart_problem |  |  |  |  |  |  |  |  | 0.47 |
| No | 2999 | 85.9 | 1.65 | 1.27 | 2.13 | 6.3 | 10.1 | <0.001 |  |
| Yes | 491 | 14.1 | 1.33 | 0.8 | 2.21 | 11.1 | 14.4 | 0.275 |  |

**Supplemental table 6: Subgroup analysis in the population with normal glucose metabolism stratified by TyG-AIP levels.**

| **Variable** | **Count** | **Percent** | **Point Estimate** | **Lower** | **Upper** | **TyG-AIP ≤ median** | **TyG-AIP > median** | **P value** | **P for interaction** |
| --- | --- | --- | --- | --- | --- | --- | --- | --- | --- |
| Overall | 2296 | 100 | 1.54 | 1.11 | 2.13 | 5.2 | 7.9 | 0.01 |  |
| Sex |  |  |  |  |  |  |  |  | 0.51 |
| Female | 1249 | 54.4 | 1.39 | 0.91 | 2.14 | 5.8 | 8 | 0.131 |  |
| Male | 1047 | 45.6 | 1.74 | 1.05 | 2.86 | 4.6 | 7.9 | 0.03 |  |
| Education |  |  |  |  |  |  |  |  | 0.995 |
| Below high school | 2027 | 88.3 | 1.55 | 1.1 | 2.17 | 5.5 | 8.4 | 0.012 |  |
| High school or above | 269 | 11.7 | 1.55 | 0.46 | 5.31 | 3.2 | 4.9 | 0.482 |  |
| Smoking |  |  |  |  |  |  |  |  | 0.726 |
| No | 1387 | 60.4 | 1.46 | 0.94 | 2.26 | 4.9 | 7.1 | 0.091 |  |
| Yes | 909 | 39.6 | 1.64 | 1 | 2.67 | 5.7 | 9.2 | 0.049 |  |
| Drinking |  |  |  |  |  |  |  |  | 0.161 |
| No | 1547 | 67.4 | 1.8 | 1.2 | 2.69 | 4.9 | 8.6 | 0.004 |  |
| Yes | 749 | 32.6 | 1.09 | 0.61 | 1.94 | 5.9 | 6.4 | 0.78 |  |
| Hukou |  |  |  |  |  |  |  |  | 0.971 |
| Rural | 1941 | 84.5 | 1.53 | 1.08 | 2.18 | 5.3 | 8 | 0.017 |  |
| Urban | 355 | 15.5 | 1.56 | 0.66 | 3.68 | 5 | 7.7 | 0.311 |  |
| Marital status |  |  |  |  |  |  |  |  | 0.486 |
| Married or partnered | 2039 | 88.8 | 1.69 | 1.17 | 2.45 | 4.4 | 7.3 | 0.006 |  |
| Separated | 257 | 11.2 | 1.27 | 0.63 | 2.57 | 11 | 13.5 | 0.507 |  |
| Dyslipidemia |  |  |  |  |  |  |  |  | 0.513 |
| No | 2126 | 92.6 | 1.48 | 1.04 | 2.12 | 4.7 | 7 | 0.03 |  |
| Yes | 170 | 7.4 | 1.1 | 0.48 | 2.51 | 15.1 | 16.2 | 0.822 |  |
| Diabetes |  |  |  |  |  |  |  |  | 0.057 |
| No | 2259 | 98.4 | 1.59 | 1.14 | 2.22 | 5 | 7.9 | 0.006 |  |
| Yes | 37 | 1.6 | 0.33 | 0.07 | 1.66 | 30 | 11.1 | 0.18 |  |
| BMI |  |  |  |  |  |  |  |  | 0.834 |
| Abnormal BMI | 784 | 34.1 | 1.55 | 0.9 | 2.65 | 6.1 | 9.3 | 0.113 |  |
| Normal BMI | 1512 | 65.9 | 1.44 | 0.94 | 2.18 | 4.9 | 7 | 0.091 |  |
| Age |  |  |  |  |  |  |  |  | 0.703 |
| >60 | 876 | 38.2 | 1.43 | 0.89 | 2.31 | 6.6 | 9.3 | 0.137 |  |
| 45-60 | 1420 | 61.8 | 1.63 | 1.04 | 2.56 | 4.4 | 7.1 | 0.032 |  |
| Heart_problem |  |  |  |  |  |  |  |  | 0.364 |
| No | 2029 | 88.4 | 1.37 | 0.95 | 1.97 | 5 | 6.8 | 0.088 |  |
| Yes | 267 | 11.6 | 2.06 | 0.93 | 4.59 | 7.5 | 15 | 0.077 |  |

**Supplemental table 7: Subgroup analysis in the total population stratified by TyG-AIP levels and hypertension status.**

| Variable | Count | Percent | Levels | Point Estimate | Lower | Upper | KM | P value | P for interaction |
| --- | --- | --- | --- | --- | --- | --- | --- | --- | --- |
| Overall | 5786 | 100 | TyG_AIP ≤ median and no hypertension | Reference |  |  | 4.7 |  |  |
|  |  |  | TyG_AIP > median and no hypertension | 1.84 | 1.44 | 2.36 | 8.5 | <0.001 |  |
|  |  |  | TyG_AIP ≤ median and hypertension | 1.95 | 1.43 | 2.65 | 8.9 | <0.001 |  |
|  |  |  | TyG_AIP > median and hypertension | 2.98 | 2.3 | 3.87 | 13.3 | <0.001 |  |
| Sex |  |  |  |  |  |  |  |  | 0.127 |
| Female | 3124 | 54 | TyG_AIP ≤ median and no hypertension | Reference |  |  | 5.3 |  |  |
|  |  |  | TyG_AIP > median and no hypertension | 1.48 | 1.06 | 2.06 | 7.8 | 0.022 |  |
|  |  |  | TyG_AIP ≤ median and hypertension | 1.44 | 0.92 | 2.25 | 7.6 | 0.107 |  |
|  |  |  | TyG_AIP > median and hypertension | 2.34 | 1.65 | 3.32 | 12 | <0.001 |  |
| Male | 2662 | 46 | TyG_AIP ≤ median and no hypertension | Reference |  |  | 4 |  |  |
|  |  |  | TyG_AIP > median and no hypertension | 2.41 | 1.65 | 3.52 | 9.3 | <0.001 |  |
|  |  |  | TyG_AIP ≤ median and hypertension | 2.66 | 1.72 | 4.13 | 10.3 | <0.001 |  |
|  |  |  | TyG_AIP > median and hypertension | 4.01 | 2.7 | 5.94 | 15 | <0.001 |  |
| Education |  |  |  |  |  |  |  |  | 0.036 |
| Below high school | 5153 | 89.1 | TyG_AIP ≤ median and no hypertension | Reference |  |  | 5.1 |  |  |
|  |  |  | TyG_AIP > median and no hypertension | 1.67 | 1.29 | 2.16 | 8.3 | <0.001 |  |
|  |  |  | TyG_AIP ≤ median and hypertension | 1.76 | 1.28 | 2.42 | 8.8 | 0.001 |  |
|  |  |  | TyG_AIP > median and hypertension | 2.77 | 2.12 | 3.63 | 13.4 | <0.001 |  |
| High school or above | 633 | 10.9 | TyG_AIP ≤ median and no hypertension | Reference |  |  | 1.3 |  |  |
|  |  |  | TyG_AIP > median and no hypertension | 7.37 | 2.21 | 24.54 | 9.3 | 0.001 |  |
|  |  |  | TyG_AIP ≤ median and hypertension | 8.69 | 2.17 | 34.76 | 11.1 | 0.002 |  |
|  |  |  | TyG_AIP > median and hypertension | 9.74 | 2.78 | 34.19 | 12.1 | <0.001 |  |
| Smoking |  |  |  |  |  |  |  |  | 0.384 |
| No | 3507 | 60.6 | TyG_AIP ≤ median and no hypertension | Reference |  |  | 4.9 |  |  |
|  |  |  | TyG_AIP > median and no hypertension | 1.59 | 1.15 | 2.2 | 7.7 | 0.005 |  |
|  |  |  | TyG_AIP ≤ median and hypertension | 1.55 | 1.02 | 2.37 | 7.5 | 0.04 |  |
|  |  |  | TyG_AIP > median and hypertension | 2.68 | 1.92 | 3.74 | 12.6 | <0.001 |  |
| Yes | 2279 | 39.4 | TyG_AIP ≤ median and no hypertension | Reference |  |  | 4.4 |  |  |
|  |  |  | TyG_AIP > median and no hypertension | 2.28 | 1.54 | 3.39 | 9.7 | <0.001 |  |
|  |  |  | TyG_AIP ≤ median and hypertension | 2.58 | 1.62 | 4.09 | 10.9 | <0.001 |  |
|  |  |  | TyG_AIP > median and hypertension | 3.53 | 2.32 | 5.37 | 14.5 | <0.001 |  |
| Drinking |  |  |  |  |  |  |  |  | 0.653 |
| No | 3891 | 67.2 | TyG_AIP ≤ median and no hypertension | Reference |  |  | 4.8 |  |  |
|  |  |  | TyG_AIP > median and no hypertension | 1.79 | 1.32 | 2.43 | 8.4 | <0.001 |  |
|  |  |  | TyG_AIP ≤ median and hypertension | 1.81 | 1.22 | 2.67 | 8.4 | 0.003 |  |
|  |  |  | TyG_AIP > median and hypertension | 2.69 | 1.95 | 3.71 | 12.3 | <0.001 |  |
| Yes | 1895 | 32.8 | TyG_AIP ≤ median and no hypertension | Reference |  |  | 4.5 |  |  |
|  |  |  | TyG_AIP > median and no hypertension | 1.94 | 1.26 | 3 | 8.6 | 0.003 |  |
|  |  |  | TyG_AIP ≤ median and hypertension | 2.22 | 1.34 | 3.68 | 9.9 | 0.002 |  |
|  |  |  | TyG_AIP > median and hypertension | 3.71 | 2.38 | 5.8 | 15.8 | <0.001 |  |
| Hukou |  |  |  |  |  |  |  |  | 0.545 |
| Rural | 4736 | 81.9 | TyG_AIP ≤ median and no hypertension | Reference |  |  | 4.6 |  |  |
|  |  |  | TyG_AIP > median and no hypertension | 1.95 | 1.48 | 2.57 | 8.7 | <0.001 |  |
|  |  |  | TyG_AIP ≤ median and hypertension | 2.05 | 1.47 | 2.87 | 9.1 | <0.001 |  |
|  |  |  | TyG_AIP > median and hypertension | 2.93 | 2.18 | 3.93 | 12.8 | <0.001 |  |
| Urban | 1050 | 18.1 | TyG_AIP ≤ median and no hypertension | Reference |  |  | 5.3 |  |  |
|  |  |  | TyG_AIP > median and no hypertension | 1.41 | 0.79 | 2.55 | 7.5 | 0.248 |  |
|  |  |  | TyG_AIP ≤ median and hypertension | 1.48 | 0.66 | 3.29 | 7.8 | 0.339 |  |
|  |  |  | TyG_AIP > median and hypertension | 3.03 | 1.7 | 5.4 | 15.3 | <0.001 |  |
| Marital status |  |  |  |  |  |  |  |  | 0.583 |
| Married or partnered | 5077 | 87.7 | TyG_AIP ≤ median and no hypertension | Reference |  |  | 4.3 |  |  |
|  |  |  | TyG_AIP > median and no hypertension | 1.97 | 1.5 | 2.58 | 8.4 | <0.001 |  |
|  |  |  | TyG_AIP ≤ median and hypertension | 1.97 | 1.39 | 2.79 | 8.3 | <0.001 |  |
|  |  |  | TyG_AIP > median and hypertension | 3.14 | 2.36 | 4.18 | 13 | <0.001 |  |
| Separated | 709 | 12.3 | TyG_AIP ≤ median and no hypertension | Reference |  |  | 7.7 |  |  |
|  |  |  | TyG_AIP > median and no hypertension | 1.22 | 0.63 | 2.36 | 9.3 | 0.56 |  |
|  |  |  | TyG_AIP ≤ median and hypertension | 1.5 | 0.76 | 2.93 | 11.3 | 0.24 |  |
|  |  |  | TyG_AIP > median and hypertension | 2.08 | 1.1 | 3.91 | 15.2 | 0.024 |  |
| Dyslipidemia |  |  |  |  |  |  |  |  | 0.859 |
| No | 5164 | 89.2 | TyG_AIP ≤ median and no hypertension | Reference |  |  | 4.4 |  |  |
|  |  |  | TyG_AIP > median and no hypertension | 1.74 | 1.33 | 2.28 | 7.6 | <0.001 |  |
|  |  |  | TyG_AIP ≤ median and hypertension | 1.82 | 1.3 | 2.55 | 7.9 | <0.001 |  |
|  |  |  | TyG_AIP > median and hypertension | 2.54 | 1.89 | 3.42 | 10.9 | <0.001 |  |
| Yes | 622 | 10.8 | TyG_AIP ≤ median and no hypertension | Reference |  |  | 8.7 |  |  |
|  |  |  | TyG_AIP > median and no hypertension | 1.72 | 0.87 | 3.38 | 14.5 | 0.117 |  |
|  |  |  | TyG_AIP ≤ median and hypertension | 2.35 | 1.05 | 5.24 | 19.1 | 0.037 |  |
|  |  |  | TyG_AIP > median and hypertension | 2.95 | 1.52 | 5.71 | 23.5 | 0.001 |  |
| Diabetes |  |  |  |  |  |  |  |  | 0.883 |
| No | 5399 | 93.3 | TyG_AIP ≤ median and no hypertension | Reference |  |  | 4.7 |  |  |
|  |  |  | TyG_AIP > median and no hypertension | 1.8 | 1.39 | 2.32 | 8.2 | <0.001 |  |
|  |  |  | TyG_AIP ≤ median and hypertension | 1.92 | 1.4 | 2.64 | 8.8 | <0.001 |  |
|  |  |  | TyG_AIP > median and hypertension | 2.8 | 2.12 | 3.68 | 12.5 | <0.001 |  |
| Yes | 387 | 6.7 | TyG_AIP ≤ median and no hypertension | Reference |  |  | 4.8 |  |  |
|  |  |  | TyG_AIP > median and no hypertension | 2.37 | 0.79 | 7.08 | 11 | 0.123 |  |
|  |  |  | TyG_AIP ≤ median and hypertension | 2.52 | 0.63 | 10.09 | 11.4 | 0.191 |  |
|  |  |  | TyG_AIP > median and hypertension | 4.26 | 1.47 | 12.3 | 18.7 | 0.008 |  |
| BMIc |  |  |  |  |  |  |  |  | 0.73 |
| Abnormal BMI | 2294 | 39.6 | TyG_AIP ≤ median and no hypertension | Reference |  |  | 5.3 |  |  |
|  |  |  | TyG_AIP > median and no hypertension | 1.71 | 1.13 | 2.58 | 8.8 | 0.011 |  |
|  |  |  | TyG_AIP ≤ median and hypertension | 1.77 | 1.06 | 2.97 | 9.2 | 0.03 |  |
|  |  |  | TyG_AIP > median and hypertension | 3.02 | 2 | 4.55 | 15.1 | <0.001 |  |
| Normal BMI | 3492 | 60.4 | TyG_AIP ≤ median and no hypertension | Reference |  |  | 4.5 |  |  |
|  |  |  | TyG_AIP > median and no hypertension | 1.86 | 1.36 | 2.57 | 8.1 | <0.001 |  |
|  |  |  | TyG_AIP ≤ median and hypertension | 2.02 | 1.38 | 2.97 | 8.8 | <0.001 |  |
|  |  |  | TyG_AIP > median and hypertension | 2.6 | 1.8 | 3.75 | 11.1 | <0.001 |  |
| Age |  |  |  |  |  |  |  |  | 0.133 |
| 45-60 | 3437 | 59.4 | TyG_AIP ≤ median and no hypertension | Reference |  |  | 3.9 |  |  |
|  |  |  | TyG_AIP > median and no hypertension | 1.85 | 1.32 | 2.6 | 7.1 | <0.001 |  |
|  |  |  | TyG_AIP ≤ median and hypertension | 2.25 | 1.43 | 3.53 | 8.5 | <0.001 |  |
|  |  |  | TyG_AIP > median and hypertension | 3.63 | 2.53 | 5.22 | 13.4 | <0.001 |  |
| >60 | 2349 | 40.6 | TyG_AIP ≤ median and no hypertension | Reference |  |  | 6.1 |  |  |
|  |  |  | TyG_AIP > median and no hypertension | 1.87 | 1.3 | 2.7 | 11 | 0.001 |  |
|  |  |  | TyG_AIP ≤ median and hypertension | 1.55 | 1.01 | 2.37 | 9.3 | 0.043 |  |
|  |  |  | TyG_AIP > median and hypertension | 2.26 | 1.55 | 3.29 | 13.2 | <0.001 |  |
| Heart_problem |  |  |  |  |  |  |  |  | 0.902 |
| No | 5028 | 86.9 | TyG_AIP ≤ median and no hypertension | Reference |  |  | 4.3 |  |  |
|  |  |  | TyG_AIP > median and no hypertension | 1.85 | 1.41 | 2.44 | 7.8 | <0.001 |  |
|  |  |  | TyG_AIP ≤ median and hypertension | 2.01 | 1.43 | 2.82 | 8.5 | <0.001 |  |
|  |  |  | TyG_AIP > median and hypertension | 2.91 | 2.17 | 3.9 | 12 | <0.001 |  |
| Yes | 758 | 13.1 | TyG_AIP ≤ median and no hypertension | Reference |  |  | 8.2 |  |  |
|  |  |  | TyG_AIP > median and no hypertension | 1.5 | 0.84 | 2.69 | 12 | 0.171 |  |
|  |  |  | TyG_AIP ≤ median and hypertension | 1.52 | 0.72 | 3.18 | 12 | 0.269 |  |
|  |  |  | TyG_AIP > median and hypertension | 2.55 | 1.42 | 4.57 | 19.6 | 0.002 |  |

**Supplemental table 8: Subgroup analysis in the population with abnormal glucose metabolism stratified by TyG-AIP levels and hypertension status.**

| **Variable** | **Count** | **Percent** | **Levels** | **Point Estimate** | **Lower** | **Upper** | **KM** | **P value** | **P for interaction** |
| --- | --- | --- | --- | --- | --- | --- | --- | --- | --- |
| Overall | 3490 | 100 | TyG_AIP ≤ median and no hypertension | Reference |  |  | 5.8 |  |  |
|  |  |  | TyG_AIP > median and no hypertension | 1.59 | 1.17 | 2.16 | 9 | 0.003 |  |
|  |  |  | TyG_AIP ≤ median and hypertension | 1.66 | 1.16 | 2.39 | 9.4 | 0.006 |  |
|  |  |  | TyG_AIP > median and hypertension | 2.52 | 1.85 | 3.44 | 13.9 | <0.001 |  |
| Sex |  |  |  |  |  |  |  |  | 0.336 |
| Female | 1875 | 53.7 | TyG_AIP ≤ median and no hypertension | Reference |  |  | 5.8 |  |  |
|  |  |  | TyG_AIP > median and no hypertension | 1.41 | 0.92 | 2.17 | 8.1 | 0.114 |  |
|  |  |  | TyG_AIP ≤ median and hypertension | 1.69 | 1.01 | 2.82 | 9.6 | 0.044 |  |
|  |  |  | TyG_AIP > median and hypertension | 2 | 1.28 | 3.12 | 11.2 | 0.002 |  |
| Male | 1615 | 46.3 | TyG_AIP ≤ median and no hypertension | Reference |  |  | 5.8 |  |  |
|  |  |  | TyG_AIP > median and no hypertension | 1.83 | 1.18 | 2.82 | 10.3 | 0.007 |  |
|  |  |  | TyG_AIP ≤ median and hypertension | 1.64 | 0.97 | 2.75 | 9.3 | 0.063 |  |
|  |  |  | TyG_AIP > median and hypertension | 3.26 | 2.11 | 5.04 | 17.6 | <0.001 |  |
| Education |  |  |  |  |  |  |  |  | 0.479 |
| Below high school | 3126 | 89.6 | TyG_AIP ≤ median and no hypertension | Reference |  |  | 6 |  |  |
|  |  |  | TyG_AIP > median and no hypertension | 1.47 | 1.06 | 2.03 | 8.6 | 0.02 |  |
|  |  |  | TyG_AIP ≤ median and hypertension | 1.64 | 1.13 | 2.39 | 9.6 | 0.01 |  |
|  |  |  | TyG_AIP > median and hypertension | 2.43 | 1.75 | 3.37 | 13.9 | <0.001 |  |
| High school or above | 364 | 10.4 | TyG_AIP ≤ median and no hypertension | Reference |  |  | 4.1 |  |  |
|  |  |  | TyG_AIP > median and no hypertension | 3.05 | 1.12 | 8.33 | 12 | 0.029 |  |
|  |  |  | TyG_AIP ≤ median and hypertension | 1.83 | 0.44 | 7.66 | 7.5 | 0.408 |  |
|  |  |  | TyG_AIP > median and hypertension | 3.68 | 1.26 | 10.78 | 14.3 | 0.017 |  |
| Smoking |  |  |  |  |  |  |  |  | 0.784 |
| No | 2120 | 60.7 | TyG_AIP ≤ median and no hypertension | Reference |  |  | 5.5 |  |  |
|  |  |  | TyG_AIP > median and no hypertension | 1.55 | 1.04 | 2.33 | 8.4 | 0.033 |  |
|  |  |  | TyG_AIP ≤ median and hypertension | 1.85 | 1.15 | 2.97 | 9.9 | 0.011 |  |
|  |  |  | TyG_AIP > median and hypertension | 2.44 | 1.62 | 3.68 | 12.9 | <0.001 |  |
| Yes | 1370 | 39.3 | TyG_AIP ≤ median and no hypertension | Reference |  |  | 6.2 |  |  |
|  |  |  | TyG_AIP > median and no hypertension | 1.66 | 1.04 | 2.64 | 10 | 0.032 |  |
|  |  |  | TyG_AIP ≤ median and hypertension | 1.43 | 0.81 | 2.54 | 8.8 | 0.216 |  |
|  |  |  | TyG_AIP > median and hypertension | 2.68 | 1.66 | 4.32 | 15.7 | <0.001 |  |
| Drinking |  |  |  |  |  |  |  |  | 0.461 |
| No | 2344 | 67.2 | TyG_AIP ≤ median and no hypertension | Reference |  |  | 5.3 |  |  |
|  |  |  | TyG_AIP > median and no hypertension | 1.78 | 1.22 | 2.62 | 9.1 | 0.003 |  |
|  |  |  | TyG_AIP ≤ median and hypertension | 1.82 | 1.15 | 2.9 | 9.3 | 0.011 |  |
|  |  |  | TyG_AIP > median and hypertension | 2.43 | 1.63 | 3.63 | 12.3 | <0.001 |  |
| Yes | 1146 | 32.8 | TyG_AIP ≤ median and no hypertension | Reference |  |  | 6.8 |  |  |
|  |  |  | TyG_AIP > median and no hypertension | 1.31 | 0.79 | 2.18 | 8.8 | 0.301 |  |
|  |  |  | TyG_AIP ≤ median and hypertension | 1.43 | 0.79 | 2.58 | 9.6 | 0.232 |  |
|  |  |  | TyG_AIP > median and hypertension | 2.81 | 1.71 | 4.61 | 17.9 | <0.001 |  |
| Hukou |  |  |  |  |  |  |  |  | 0.118 |
| Rural | 2795 | 80.1 | TyG_AIP ≤ median and no hypertension | Reference |  |  | 5.5 |  |  |
|  |  |  | TyG_AIP > median and no hypertension | 1.76 | 1.25 | 2.48 | 9.4 | 0.001 |  |
|  |  |  | TyG_AIP ≤ median and hypertension | 1.91 | 1.28 | 2.84 | 10.2 | 0.001 |  |
|  |  |  | TyG_AIP > median and hypertension | 2.43 | 1.7 | 3.47 | 12.8 | <0.001 |  |
| Urban | 695 | 19.9 | TyG_AIP ≤ median and no hypertension | Reference |  |  | 7.2 |  |  |
|  |  |  | TyG_AIP > median and no hypertension | 1.06 | 0.54 | 2.08 | 7.6 | 0.872 |  |
|  |  |  | TyG_AIP ≤ median and hypertension | 0.84 | 0.33 | 2.16 | 6.1 | 0.717 |  |
|  |  |  | TyG_AIP > median and hypertension | 2.66 | 1.4 | 5.05 | 18 | 0.003 |  |
| Marital status |  |  |  |  |  |  |  |  | 0.87 |
| Married or partnered | 3038 | 87 | TyG_AIP ≤ median and no hypertension | Reference |  |  | 5.9 |  |  |
|  |  |  | TyG_AIP > median and no hypertension | 1.58 | 1.14 | 2.18 | 9.1 | 0.006 |  |
|  |  |  | TyG_AIP ≤ median and hypertension | 1.55 | 1.03 | 2.32 | 8.9 | 0.034 |  |
|  |  |  | TyG_AIP > median and hypertension | 2.44 | 1.75 | 3.4 | 13.7 | <0.001 |  |
| Separated | 452 | 13 | TyG_AIP ≤ median and no hypertension | Reference |  |  | 5.2 |  |  |
|  |  |  | TyG_AIP > median and no hypertension | 1.73 | 0.67 | 4.47 | 8.7 | 0.255 |  |
|  |  |  | TyG_AIP ≤ median and hypertension | 2.27 | 0.9 | 5.78 | 11.3 | 0.084 |  |
|  |  |  | TyG_AIP > median and hypertension | 3.16 | 1.26 | 7.92 | 15.3 | 0.014 |  |
| Dyslipidemia |  |  |  |  |  |  |  |  | 0.472 |
| No | 3038 | 87 | TyG_AIP ≤ median and no hypertension | Reference |  |  | 5.6 |  |  |
|  |  |  | TyG_AIP > median and no hypertension | 1.45 | 1.04 | 2.02 | 8 | 0.029 |  |
|  |  |  | TyG_AIP ≤ median and hypertension | 1.44 | 0.96 | 2.15 | 8 | 0.08 |  |
|  |  |  | TyG_AIP > median and hypertension | 2.06 | 1.44 | 2.94 | 11.2 | <0.001 |  |
| Yes | 452 | 13 | TyG_AIP ≤ median and no hypertension | Reference |  |  | 7.6 |  |  |
|  |  |  | TyG_AIP > median and no hypertension | 1.99 | 0.86 | 4.61 | 14.6 | 0.11 |  |
|  |  |  | TyG_AIP ≤ median and hypertension | 3.01 | 1.18 | 7.63 | 21.1 | 0.021 |  |
|  |  |  | TyG_AIP > median and hypertension | 3.41 | 1.51 | 7.71 | 23.7 | 0.003 |  |
| Diabetes |  |  |  |  |  |  |  |  | 0.139 |
| No | 3140 | 90 | TyG_AIP ≤ median and no hypertension | Reference |  |  | 6 |  |  |
|  |  |  | TyG_AIP > median and no hypertension | 1.47 | 1.07 | 2.02 | 8.7 | 0.018 |  |
|  |  |  | TyG_AIP ≤ median and hypertension | 1.63 | 1.12 | 2.36 | 9.6 | 0.011 |  |
|  |  |  | TyG_AIP > median and hypertension | 2.2 | 1.57 | 3.07 | 12.7 | <0.001 |  |
| Yes | 350 | 10 | TyG_AIP ≤ median and no hypertension | Reference |  |  | 2.5 |  |  |
|  |  |  | TyG_AIP > median and no hypertension | 4.86 | 1.11 | 21.26 | 11.8 | 0.036 |  |
|  |  |  | TyG_AIP ≤ median and hypertension | 3.1 | 0.52 | 18.56 | 7.5 | 0.215 |  |
|  |  |  | TyG_AIP > median and hypertension | 8.85 | 2.08 | 37.77 | 20.2 | 0.003 |  |
| BMI |  |  |  |  |  |  |  |  | 0.617 |
| Abnormal BMI | 1510 | 43.3 | TyG_AIP ≤ median and no hypertension | Reference |  |  | 5.9 |  |  |
|  |  |  | TyG_AIP > median and no hypertension | 1.56 | 0.94 | 2.58 | 9 | 0.086 |  |
|  |  |  | TyG_AIP ≤ median and hypertension | 1.83 | 1.02 | 3.28 | 10.5 | 0.044 |  |
|  |  |  | TyG_AIP > median and hypertension | 2.83 | 1.74 | 4.62 | 15.8 | <0.001 |  |
| Normal BMI | 1980 | 56.7 | TyG_AIP ≤ median and no hypertension | Reference |  |  | 5.8 |  |  |
|  |  |  | TyG_AIP > median and no hypertension | 1.61 | 1.09 | 2.39 | 9.1 | 0.017 |  |
|  |  |  | TyG_AIP ≤ median and hypertension | 1.53 | 0.95 | 2.47 | 8.7 | 0.078 |  |
|  |  |  | TyG_AIP > median and hypertension | 2.03 | 1.29 | 3.2 | 11.2 | 0.002 |  |
| Age |  |  |  |  |  |  |  |  | 0.085 |
| 45-60 | 1473 | 42.2 | TyG_AIP ≤ median and no hypertension | Reference |  |  | 7.2 |  |  |
|  |  |  | TyG_AIP > median and no hypertension | 1.8 | 1.16 | 2.79 | 12.6 | 0.009 |  |
|  |  |  | TyG_AIP ≤ median and hypertension | 1.41 | 0.86 | 2.29 | 10 | 0.169 |  |
|  |  |  | TyG_AIP > median and hypertension | 1.87 | 1.19 | 2.95 | 13.1 | 0.007 |  |
| >60 | 2017 | 57.8 | TyG_AIP ≤ median and no hypertension | Reference |  |  | 4.9 |  |  |
|  |  |  | TyG_AIP > median and no hypertension | 1.48 | 0.97 | 2.26 | 7.1 | 0.07 |  |
|  |  |  | TyG_AIP ≤ median and hypertension | 1.79 | 1.02 | 3.14 | 8.5 | 0.043 |  |
|  |  |  | TyG_AIP > median and hypertension | 3.18 | 2.07 | 4.87 | 14.7 | <0.001 |  |
| Heart_problem |  |  |  |  |  |  |  |  | 0.849 |
| No | 2999 | 85.9 | TyG_AIP ≤ median and no hypertension | Reference |  |  | 5.3 |  |  |
|  |  |  | TyG_AIP > median and no hypertension | 1.62 | 1.15 | 2.27 | 8.4 | 0.006 |  |
|  |  |  | TyG_AIP ≤ median and hypertension | 1.7 | 1.13 | 2.54 | 8.8 | 0.011 |  |
|  |  |  | TyG_AIP > median and hypertension | 2.61 | 1.84 | 3.7 | 13.2 | <0.001 |  |
| Yes | 491 | 14.1 | TyG_AIP ≤ median and no hypertension | Reference |  |  | 10 |  |  |
|  |  |  | TyG_AIP > median and no hypertension | 1.29 | 0.64 | 2.57 | 12.5 | 0.477 |  |
|  |  |  | TyG_AIP ≤ median and hypertension | 1.35 | 0.59 | 3.07 | 13 | 0.479 |  |
|  |  |  | TyG_AIP > median and hypertension | 1.81 | 0.9 | 3.64 | 17.2 | 0.096 |  |

**Supplemental table 9: Subgroup analysis in the population with normal glucose metabolism stratified by TyG-AIP levels and hypertension status.**

| **Variable** | **Count** | **Percent** | **Levels** | **Point Estimate** | **Lower** | **Upper** | **KM** | **P value** | **P for interaction** |
| --- | --- | --- | --- | --- | --- | --- | --- | --- | --- |
| Overall | 2296 | 100 | TyG_AIP ≤ median and no hypertension | Reference |  |  | 3.9 |  |  |
|  |  |  | TyG_AIP > median and no hypertension | 1.75 | 1.15 | 2.66 | 6.8 | 0.009 |  |
|  |  |  | TyG_AIP ≤ median and hypertension | 2.5 | 1.5 | 4.18 | 9.6 | <0.001 |  |
|  |  |  | TyG_AIP > median and hypertension | 2.88 | 1.8 | 4.62 | 11 | <0.001 |  |
| Sex |  |  |  |  |  |  |  |  | 0.13 |
| Female | 1249 | 54.4 | TyG_AIP ≤ median and no hypertension | Reference |  |  | 5.3 |  |  |
|  |  |  | TyG_AIP > median and no hypertension | 1.3 | 0.77 | 2.2 | 6.8 | 0.32 |  |
|  |  |  | TyG_AIP ≤ median and hypertension | 1.46 | 0.7 | 3.05 | 7.6 | 0.308 |  |
|  |  |  | TyG_AIP > median and hypertension | 2.17 | 1.19 | 3.94 | 11.1 | 0.011 |  |
| Male | 1047 | 45.6 | TyG_AIP ≤ median and no hypertension | Reference |  |  | 2.4 |  |  |
|  |  |  | TyG_AIP > median and no hypertension | 2.85 | 1.37 | 5.94 | 6.8 | 0.005 |  |
|  |  |  | TyG_AIP ≤ median and hypertension | 4.99 | 2.24 | 11.12 | 11.6 | <0.001 |  |
|  |  |  | TyG_AIP > median and hypertension | 4.67 | 2.1 | 10.4 | 10.8 | <0.001 |  |
| Education |  |  |  |  |  |  |  |  | 0.011 |
| Below high school | 2027 | 88.3 | TyG_AIP ≤ median and no hypertension | Reference |  |  | 4.5 |  |  |
|  |  |  | TyG_AIP > median and no hypertension | 1.61 | 1.05 | 2.47 | 7.1 | 0.03 |  |
|  |  |  | TyG_AIP ≤ median and hypertension | 1.97 | 1.15 | 3.38 | 8.6 | 0.014 |  |
|  |  |  | TyG_AIP > median and hypertension | 2.67 | 1.65 | 4.31 | 11.6 | <0.001 |  |
| High school or above | 269 | 11.7 | TyG_AIP ≤ median and no hypertension | Reference |  |  | 0 |  |  |
|  |  |  | TyG_AIP > median and no hypertension | 230052200.4 | 0 | Inf | 4.5 | 0.998 |  |
|  |  |  | TyG_AIP ≤ median and hypertension | 1227719062 | 0 | Inf | 22.2 | 0.998 |  |
|  |  |  | TyG_AIP > median and hypertension | 308958417 | 0 | Inf | 6.1 | 0.998 |  |
| Smoking |  |  |  |  |  |  |  |  | 0.215 |
| No | 1387 | 60.4 | TyG_AIP ≤ median and no hypertension | Reference |  |  | 4.4 |  |  |
|  |  |  | TyG_AIP > median and no hypertension | 1.45 | 0.85 | 2.46 | 6.3 | 0.168 |  |
|  |  |  | TyG_AIP ≤ median and hypertension | 1.54 | 0.74 | 3.22 | 6.7 | 0.252 |  |
|  |  |  | TyG_AIP > median and hypertension | 2.11 | 1.13 | 3.92 | 9.1 | 0.019 |  |
| Yes | 909 | 39.6 | TyG_AIP ≤ median and no hypertension | Reference |  |  | 3.2 |  |  |
|  |  |  | TyG_AIP > median and no hypertension | 2.38 | 1.17 | 4.83 | 7.5 | 0.017 |  |
|  |  |  | TyG_AIP ≤ median and hypertension | 4.39 | 2.01 | 9.55 | 13.4 | <0.001 |  |
|  |  |  | TyG_AIP > median and hypertension | 4.56 | 2.14 | 9.74 | 13.8 | <0.001 |  |
| Drinking |  |  |  |  |  |  |  |  | 0.44 |
| No | 1547 | 67.4 | TyG_AIP ≤ median and no hypertension | Reference |  |  | 3.9 |  |  |
|  |  |  | TyG_AIP > median and no hypertension | 1.99 | 1.19 | 3.32 | 7.5 | 0.009 |  |
|  |  |  | TyG_AIP ≤ median and hypertension | 2.18 | 1.12 | 4.26 | 8.2 | 0.023 |  |
|  |  |  | TyG_AIP > median and hypertension | 3.07 | 1.73 | 5.45 | 11.4 | <0.001 |  |
| Yes | 749 | 32.6 | TyG_AIP ≤ median and no hypertension | Reference |  |  | 4.1 |  |  |
|  |  |  | TyG_AIP > median and no hypertension | 1.25 | 0.58 | 2.7 | 5.1 | 0.567 |  |
|  |  |  | TyG_AIP ≤ median and hypertension | 3.08 | 1.38 | 6.87 | 12.2 | 0.006 |  |
|  |  |  | TyG_AIP > median and hypertension | 2.5 | 1.07 | 5.84 | 10 | 0.035 |  |
| Hukou |  |  |  |  |  |  |  |  | 0.685 |
| Rural | 1941 | 84.5 | TyG_AIP ≤ median and no hypertension | Reference |  |  | 4.1 |  |  |
|  |  |  | TyG_AIP > median and no hypertension | 1.68 | 1.07 | 2.63 | 6.8 | 0.025 |  |
|  |  |  | TyG_AIP ≤ median and hypertension | 2.23 | 1.28 | 3.88 | 8.9 | 0.005 |  |
|  |  |  | TyG_AIP > median and hypertension | 2.84 | 1.7 | 4.73 | 11.2 | <0.001 |  |
| Urban | 355 | 15.5 | TyG_AIP ≤ median and no hypertension | Reference |  |  | 3 |  |  |
|  |  |  | TyG_AIP > median and no hypertension | 2.3 | 0.71 | 7.45 | 6.8 | 0.167 |  |
|  |  |  | TyG_AIP ≤ median and hypertension | 5.47 | 1.37 | 21.88 | 15.4 | 0.016 |  |
|  |  |  | TyG_AIP > median and hypertension | 3.39 | 0.96 | 12.01 | 9.8 | 0.059 |  |
| Marital status |  |  |  |  |  |  |  |  | 0.486 |
| Married or partnered | 2039 | 88.8 | TyG_AIP ≤ median and no hypertension | Reference |  |  | 3.3 |  |  |
|  |  |  | TyG_AIP > median and no hypertension | 1.97 | 1.22 | 3.17 | 6.3 | 0.005 |  |
|  |  |  | TyG_AIP ≤ median and hypertension | 2.79 | 1.53 | 5.1 | 8.8 | 0.001 |  |
|  |  |  | TyG_AIP > median and hypertension | 3.26 | 1.9 | 5.58 | 10.3 | <0.001 |  |
| Separated | 257 | 11.2 | TyG_AIP ≤ median and no hypertension | Reference |  |  | 10.1 |  |  |
|  |  |  | TyG_AIP > median and no hypertension | 1.26 | 0.49 | 3.26 | 12.5 | 0.636 |  |
|  |  |  | TyG_AIP ≤ median and hypertension | 1.22 | 0.46 | 3.28 | 12.3 | 0.691 |  |
|  |  |  | TyG_AIP > median and hypertension | 1.55 | 0.58 | 4.15 | 14.9 | 0.387 |  |
| Dyslipidemia |  |  |  |  |  |  |  |  | 0.821 |
| No | 2126 | 92.6 | TyG_AIP ≤ median and no hypertension | Reference |  |  | 3.5 |  |  |
|  |  |  | TyG_AIP > median and no hypertension | 1.76 | 1.11 | 2.79 | 6.2 | 0.015 |  |
|  |  |  | TyG_AIP ≤ median and hypertension | 2.62 | 1.51 | 4.53 | 9 | 0.001 |  |
|  |  |  | TyG_AIP > median and hypertension | 2.73 | 1.61 | 4.64 | 9.4 | <0.001 |  |
| Yes | 170 | 7.4 | TyG_AIP ≤ median and no hypertension | Reference |  |  | 13.5 |  |  |
|  |  |  | TyG_AIP > median and no hypertension | 1.02 | 0.35 | 2.99 | 13.5 | 0.969 |  |
|  |  |  | TyG_AIP ≤ median and hypertension | 1.46 | 0.35 | 6.13 | 18.8 | 0.601 |  |
|  |  |  | TyG_AIP > median and hypertension | 1.66 | 0.56 | 4.94 | 20.9 | 0.366 |  |
| Diabetes |  |  |  |  |  |  |  |  | 0.023 |
| No | 2259 | 98.4 | TyG_AIP ≤ median and no hypertension | Reference |  |  | 3.6 |  |  |
|  |  |  | TyG_AIP > median and no hypertension | 1.93 | 1.25 | 2.97 | 6.9 | 0.003 |  |
|  |  |  | TyG_AIP ≤ median and hypertension | 2.72 | 1.61 | 4.6 | 9.6 | <0.001 |  |
|  |  |  | TyG_AIP > median and hypertension | 2.97 | 1.81 | 4.86 | 10.4 | <0.001 |  |
| Yes | 37 | 1.6 | TyG_AIP ≤ median and no hypertension | Reference |  |  | 33.3 |  |  |
|  |  |  | TyG_AIP > median and no hypertension | 0 | 0 | Inf | 0 | 0.999 |  |
|  |  |  | TyG_AIP ≤ median and hypertension | 0 | 0 | Inf | 0 | 1 |  |
|  |  |  | TyG_AIP > median and hypertension | 0.65 | 0.13 | 3.2 | 23.1 | 0.593 |  |
| BMI |  |  |  |  |  |  |  |  | 0.368 |
| Abnormal BMI | 784 | 34.1 | TyG_AIP ≤ median and no hypertension | Reference |  |  | 5.8 |  |  |
|  |  |  | TyG_AIP > median and no hypertension | 1.33 | 0.69 | 2.55 | 7.7 | 0.398 |  |
|  |  |  | TyG_AIP ≤ median and hypertension | 1.21 | 0.44 | 3.37 | 7 | 0.71 |  |
|  |  |  | TyG_AIP > median and hypertension | 2.29 | 1.15 | 4.57 | 12.9 | 0.019 |  |
| Normal BMI | 1512 | 65.9 | TyG_AIP ≤ median and no hypertension | Reference |  |  | 3.2 |  |  |
|  |  |  | TyG_AIP > median and no hypertension | 1.95 | 1.13 | 3.38 | 6.2 | 0.017 |  |
|  |  |  | TyG_AIP ≤ median and hypertension | 3.37 | 1.83 | 6.21 | 10.5 | <0.001 |  |
|  |  |  | TyG_AIP > median and hypertension | 2.94 | 1.51 | 5.69 | 9.2 | 0.001 |  |
| Age |  |  |  |  |  |  |  |  | 0.944 |
| 45-60 | 876 | 38.2 | TyG_AIP ≤ median and no hypertension | Reference |  |  | 5 |  |  |
|  |  |  | TyG_AIP > median and no hypertension | 1.69 | 0.88 | 3.23 | 8.2 | 0.115 |  |
|  |  |  | TyG_AIP ≤ median and hypertension | 2.17 | 1.05 | 4.5 | 10.4 | 0.037 |  |
|  |  |  | TyG_AIP > median and hypertension | 2.4 | 1.21 | 4.76 | 11.4 | 0.012 |  |
| >60 | 1420 | 61.8 | TyG_AIP ≤ median and no hypertension | Reference |  |  | 3.4 |  |  |
|  |  |  | TyG_AIP > median and no hypertension | 1.8 | 1.04 | 3.13 | 6.1 | 0.037 |  |
|  |  |  | TyG_AIP ≤ median and hypertension | 2.6 | 1.24 | 5.42 | 8.7 | 0.011 |  |
|  |  |  | TyG_AIP > median and hypertension | 3.17 | 1.64 | 6.11 | 10.5 | 0.001 |  |
| Heart_problem |  |  |  |  |  |  |  |  | 0.492 |
| No | 2029 | 88.4 | TyG_AIP ≤ median and no hypertension | Reference |  |  | 3.7 |  |  |
|  |  |  | TyG_AIP > median and no hypertension | 1.68 | 1.06 | 2.67 | 6.2 | 0.027 |  |
|  |  |  | TyG_AIP ≤ median and hypertension | 2.59 | 1.49 | 4.49 | 9.4 | 0.001 |  |
|  |  |  | TyG_AIP > median and hypertension | 2.33 | 1.34 | 4.03 | 8.4 | 0.003 |  |
| Yes | 267 | 11.6 | TyG_AIP ≤ median and no hypertension | Reference |  |  | 6.2 |  |  |
|  |  |  | TyG_AIP > median and no hypertension | 1.77 | 0.62 | 5.03 | 10.8 | 0.283 |  |
|  |  |  | TyG_AIP ≤ median and hypertension | 1.92 | 0.46 | 8.05 | 11.5 | 0.37 |  |
|  |  |  | TyG_AIP > median and hypertension | 4.33 | 1.53 | 12.3 | 24.5 | 0.006 |  |

**Supplemental Table 10. Summary of Interaction, Joint Effect, and Reclassification Analyses of TyG-AIP and Hypertension on Stroke Risk.** AIP, atherogenic index of plasma; CI, confidence interval; HR, hazard ratio; HTN, hypertension; NRI, Net Reclassification Index; TyG, triglyceride-glucose index; PDM, population with dysglycemia; NDM, population with normoglycemia.

| **Population** | **Analysis Type** | **Key Metric** | **Result (HR or NRI, 95% CI)** | **P Value** | **Primary Interpretation** |
| --- | --- | --- | --- | --- | --- |
| ****Overall**** | Multiplicative Interaction (Continuous) | TyG-AIP × HBP interaction term | ****0.947 (0.904 – 0.992)**** | ****0.022**** | Negative interaction: the risk gradient per unit TyG-AIP is attenuated in hypertensives. |
|  | Joint Exposure (Categorical) | High AIP + Hypertension (vs. Low AIP & No HTN) | ****2.981 (2.297 – 3.869)**** | ****<0.001**** | ****Highest-risk clinical phenotype****: combined exposure confers the greatest hazard. |
|  | Risk Reclassification (NRI) | Continuous NRI | ****0.193 (0.101 – 0.288)**** | ****<0.001**** | TyG-AIP adds significant incremental value for individual risk stratification. |
| ****Dysglycemia (PDM)**** | Multiplicative Interaction (Continuous) | TyG-AIP × HBP interaction term | 0.960 (0.911 – 1.012) | 0.127 | No statistically significant interaction observed. |
|  | Joint Exposure (Categorical) | High AIP + Hypertension (vs. Low AIP & No HTN) | ****2.521 (1.847 – 3.441)**** | ****<0.001**** | Combined exposure remains the strongest risk factor. |
|  | Risk Reclassification (NRI) | Continuous NRI | ****0.162 (0.045 – 0.280)**** | ****0.007**** | NRI remains positive and significant. |
| ****Normoglycemia (NDM)**** | Multiplicative Interaction (Continuous) | TyG-AIP × HBP interaction term | 0.904 (0.800 – 1.021) | 0.105 | No statistically significant interaction observed. |
|  | Joint Exposure (Categorical) | High AIP + Hypertension (vs. Low AIP & No HTN) | ****2.885 (1.799 – 4.624)**** | ****<0.001**** | Combined exposure remains the strongest risk factor. |
|  | Risk Reclassification (NRI) | Continuous NRI | ****0.190 (0.030 – 0.346)**** | ****0.020**** | NRI remains positive and significant. |

Note: The joint exposure variable was created by cross-classifying participants based on TyG-AIP level (high/low by median) and hypertension status (yes/no). The reference group for hazard ratios is “Low AIP & No Hypertension”.

****Supplemental Table 11. Net Reclassification Index (NRI) for the Improvement of Stroke Risk Prediction by Adding TyG-AIP to a Conventional Model.****

| **Population** | **N (Events)** | **Continuous NRI (95% CI)** | **P Value** |
| --- | --- | --- | --- |
| **Overall** | 5786 (460) | 0.193 (0.101 – 0.288) | <0.001 |
| **Dysglycemia (PDM)** | 3490 (309) | 0.162 (0.045 – 0.280) | 0.007 |
| **Normoglycemia (NDM)** | 2296 (151) | 0.190 (0.030 – 0.346) | 0.020 |

P value derived from the bootstrap confidence interval (CI not including 0).

****Supplemental Table 12**. Hazard Ratios for Stroke according to Baseline TyG-AIP Strata by Tertiles and Quartiles.**

| **Population** | **Group** | **Model 1 HR (95% CI)** | **Model 2 HR (95% CI)** | **Model 3 HR (95% CI)** | **P for Trend** |
| --- | --- | --- | --- | --- | --- |
| ****Total**** |  |  |  |  | ****<0.001**** |
|  | Quartile 2 (Q2) | 1.43 (1.06, 1.95)* | 1.44 (1.06, 1.96)* | 1.39 (1.02, 1.89)* |  |
|  | Quartile 3 (Q3) | 2.14 (1.61, 2.85)*** | 2.16 (1.62, 2.87)*** | 1.99 (1.48, 2.66)*** |  |
|  | Quartile 4 (Q4) | 2.28 (1.71, 3.03)*** | 2.30 (1.73, 3.06)*** | 1.93 (1.44, 2.60)*** |  |
|  | Tertile 2 (T2) | 1.72 (1.34, 2.20)*** | 1.73 (1.35, 2.22)*** | 1.65 (1.29, 2.12)*** | ****<0.001**** |
|  | Tertile 3 (T3) | 2.04 (1.60, 2.60)*** | 2.06 (1.61, 2.62)*** | 1.76 (1.36, 2.27)*** |  |
| ****PDM**** |  |  |  |  | ****<0.001**** |
|  | Quartile 2 (Q2) | 1.35 (0.94, 1.94) | 1.36 (0.95, 1.95) | 1.32 (0.92, 1.91) |  |
|  | Quartile 3 (Q3) | 1.90 (1.35, 2.68)*** | 1.92 (1.36, 2.70)*** | 1.77 (1.25, 2.50)** |  |
|  | Quartile 4 (Q4) | 2.04 (1.46, 2.87)*** | 2.05 (1.46, 2.89)*** | 1.81 (1.26, 2.59)** |  |
|  | Tertile 2 (T2) | 1.86 (1.38, 2.50)*** | 1.88 (1.39, 2.53)*** | 1.81 (1.34, 2.45)*** | ****<0.001**** |
|  | Tertile 3 (T3) | 1.95 (1.44, 2.63)*** | 1.96 (1.45, 2.65)*** | 1.73 (1.26, 2.37)** |  |
| ****NDM**** |  |  |  |  | ****<0.001**** |
|  | Quartile 2 (Q2) | 2.03 (1.19, 3.48)* | 2.03 (1.18, 3.48)* | 1.76 (1.02, 3.04)* |  |
|  | Quartile 3 (Q3) | 2.22 (1.31, 3.77)** | 2.30 (1.35, 3.92)** | 1.95 (1.13, 3.38)* |  |
|  | Quartile 4 (Q4) | 2.43 (1.44, 4.10)** | 2.45 (1.44, 4.15)** | 1.89 (1.10, 3.24)* |  |
|  | Tertile 2 (T2) | 1.97 (1.27, 3.05)** | 1.98 (1.27, 3.07)** | 1.78 (1.13, 2.80)* | ****0.001**** |
|  | Tertile 3 (T3) | 2.10 (1.36, 3.26)*** | 2.11 (1.36, 3.29)*** | 1.73 (1.10, 2.72)* |  |

Abbreviations: CI, confidence interval; HR, hazard ratio; NDM, newly diagnosed diabetes; PDM, prediabetes.

Model 1: Unadjusted.

Model 2: Adjusted for age and sex.

Model 3: Adjusted for age, sex, smoking, drinking, BMI, physical activity, family history of diabetes, and hypertension.

Reference groups: Quartile 1 (Q1) for quartile analysis; Tertile 1 (T1) for tertile analysis.

P for trend was calculated across the ordinal groups (Q1-Q4 or T1-T3).

* P < 0.05, ** P < 0.01, *** P < 0.001.

****Supplemental Table 13. Hazard ratios for key comparisons assessing the modifying effect of hypertension across study populations.** HTN, hypertension; HR, hazard ratio; CI, confidence interval.**

| **Comparison Context** | **Comparison Group vs. Reference Group** | **Total Population** | **PDM Population** | **NDM Population** |
| --- | --- | --- | --- | --- |
|  |  | ****HR (95% CI), P-value**** | ****HR (95% CI), P-value**** | ****HR (95% CI), P-value**** |
| ****Formal Interaction Test**** | Trajectory × Hypertension (Likelihood Ratio Test) | P = 0.113 | P = 0.521 | P = 0.113 |
| ****Within Hypertensive Subgroup**** | ****Medium-risk & HTN**** vs. Low-risk & HTN | 0.69 (0.27–1.79), 0.444 | ****1.32 (0.84–2.07), 0.230**** | 0.69 (0.27–1.79), 0.444 |
|  | ****High-risk & HTN**** vs. Low-risk & HTN | 0.39 (0.15–1.04), 0.059 | ****1.77 (1.11–2.82), 0.017**** | 0.39 (0.15–1.04), 0.059 |
| ****Within Non-Hypertensive Subgroup**** | ****Medium-risk & No HTN**** vs. Low-risk & No HTN | 1.25 (0.63–2.51), 0.521 | 1.16 (0.82–1.64), 0.399 | 1.25 (0.63–2.51), 0.521 |
|  | ****High-risk & No HTN**** vs. Low-risk & No HTN | 1.97 (0.99–3.92), 0.055 | 2.17 (1.48–3.20), <0.001 | 1.97 (0.99–3.92), 0.055 |
| ****Within the Same Trajectory**** | ****Low-risk & HTN**** vs. Low-risk & No HTN | ****3.38 (1.50–7.61), 0.003**** | ****1.63 (1.11–2.38), 0.013**** | ****3.38 (1.50–7.61), 0.003**** |
|  | ****Medium-risk & HTN**** vs. Medium-risk & No HTN | ****2.33 (1.00–5.42), 0.050**** | ****1.84 (1.20–2.83), 0.006**** | ****2.33 (1.00–5.42), 0.050**** |
|  | ****High-risk & HTN**** vs. High-risk & No HTN | 1.33 (0.60–2.94), 0.480 | 1.32 (0.81–2.16), 0.270 | 1.33 (0.60–2.94), 0.480 |

Note: Bold text indicates statistically significant results (P < 0.05). All hazard ratios are derived from Cox proportional hazards models. The formal interaction test assesses the significance of the multiplicative interaction term (Trajectory × Hypertension) using the likelihood ratio test.

****Supplemental Table 14**. Discriminative Performance of TyG, AIP, TyG-AIP, and Hypertension for Stroke Prediction Across Study Populations.**

| **Population** | **Model** | **AUC (95% CI)** | **Optimal Cutoff** | **Sensitivity** | **Specificity** | **PPV** | **NPV** | **P-value vs. TyG-AIP*** |
| --- | --- | --- | --- | --- | --- | --- | --- | --- |
| ****Total**** | Hypertension | 0.571 (0.547-0.594) | N/A (binary) | 71.1% | 43.0% | 9.3% | 94.6% | 0.258 |
|  | TyG | 0.586 (0.560-0.612) | 8.604 | 64.8% | 49.5% | 10.0% | 94.2% | 0.447 |
|  | AIP | 0.590 (0.564-0.616) | 0.322 | 65.4% | 49.6% | 10.1% | 94.3% | 0.287 |
|  | ****TyG-AIP**** | ****0.590 (0.565-0.616)**** | ****2.795**** | ****65.2%**** | ****50.0%**** | ****10.1%**** | ****94.3%**** | ****Reference**** |
| ****PDM**** | Hypertension | 0.563 (0.534-0.592) | N/A (binary) | 67.7% | 45.0% | 9.3% | 94.1% | 0.337 |
|  | TyG | 0.576 (0.543-0.608) | 8.609 | 75.1% | 37.2% | 10.4% | 93.9% | 0.210 |
|  | AIP | 0.584 (0.552-0.616) | 0.324 | 71.5% | 44.3% | 11.1% | 94.1% | 0.746 |
|  | ****TyG-AIP**** | ****0.584 (0.552-0.616)**** | ****3.012**** | ****69.6%**** | ****46.4%**** | ****11.2%**** | ****94.0%**** | ****Reference**** |
| ****NDM**** | Hypertension | 0.576 (0.536-0.616) | N/A (binary) | 76.1% | 39.1% | 5.3% | 96.9% | 0.800 |
|  | TyG | 0.581 (0.537-0.624) | 8.128 | 84.1% | 29.5% | 7.7% | 96.3% | 0.732 |
|  | AIP | 0.583 (0.540-0.627) | 0.160 | 80.1% | 35.4% | 8.0% | 96.2% | 0.482 |
|  | ****TyG-AIP**** | ****0.584 (0.540-0.627)**** | ****1.297**** | ****80.1%**** | ****35.2%**** | ****8.0%**** | ****96.2%**** | ****Reference**** |

AUC, area under the receiver operating characteristic curve; CI, confidence interval; PPV, positive predictive value; NPV, negative predictive value; * P-values from DeLong test for comparison of AUCs between each model and TyG-AIP within the same population.

****Supplemental Table 15**. Interaction Analysis of TyG-AIP with Glycemic Status.**

| **Variable** | **Hazard Ratio (HR)** | **95% CI** | **P-value** | **Interaction P-value** |
| --- | --- | --- | --- | --- |
| ****TyG-AIP effect by glycemic status**** |  |  |  |  |
| Normal (reference) | 1.098 | 1.035–1.165 | 0.002 | – |
| Prediabetes | 1.072* | 0.999–1.151 | 0.056 | 0.501 |
| Diabetes | 1.061* | 0.982–1.146 | 0.136 | 0.331 |
| ****Main effects**** |  |  |  |  |
| Prediabetes (vs. Normal) | 1.249 | 0.906–1.723 | 0.175 |  |
| Diabetes (vs. Normal) | 1.315 | 0.865–1.999 | 0.201 |  |

*Calculated as exp(0.0935–0.0244) for prediabetes and exp(0.0935–0.0351) for diabetes, representing the TyG-AIP effect within each subgroup.

Note: Model adjusted for age, sex, hypertension, smoking, drinking, and BMI. Interaction terms test whether the association between TyG-AIP and cardiovascular outcomes differs significantly between glycemic categories. The non-significant interaction p-values indicate no evidence of effect modification by glycemic status.
